# Leveraging deep phenotyping from health check-up cohort with 10,000 Korean individuals for phenome-wide association study of 136 traits

**DOI:** 10.1038/s41598-021-04580-2

**Published:** 2022-02-04

**Authors:** Eun Kyung Choe, Manu Shivakumar, Anurag Verma, Shefali Setia Verma, Seung Ho Choi, Joo Sung Kim, Dokyoon Kim

**Affiliations:** 1 Department of Biostatistics, Epidemiology and Informatics, Perelman School of Medicine, University of Pennsylvania, B304 Richards Building, 3700 Hamilton Walk, Philadelphia, PA 19104-6116 USA; 2 Department of Surgery, Seoul National University Hospital Healthcare System Gangnam Center, Seoul, 06236 South Korea; 3 Department of Genetics, Perelman School of Medicine, University of Pennsylvania, Philadelphia, PA 19104 USA; 4 Department of Internal Medicine, Seoul National University Hospital Healthcare System Gangnam Center, Seoul, 06236 South Korea; 5 Department of Internal Medicine and Liver Research Institute, Seoul National University College of Medicine, Seoul, 03080 South Korea; 6 Institute for Biomedical Informatics, University of Pennsylvania, Philadelphia, PA 19104 USA

**Keywords:** Genetics, Health care

## Abstract

The expanding use of the phenome-wide association study (PheWAS) faces challenges in the context of using International Classification of Diseases billing codes for phenotype definition, imbalanced study population ethnicity, and constrained application of the results in research. We performed a PheWAS utilizing 136 deep phenotypes corroborated by comprehensive health check-ups in a Korean population, along with trans-ethnic comparisons through using the UK Biobank and Biobank Japan Project. Meta-analysis with Korean and Japanese population was done. The PheWAS associated 65 phenotypes with 14,101 significant variants (*P* < 4.92 × 10–10). Network analysis, visualization of cross-phenotype mapping, and causal inference mapping with Mendelian randomization were conducted. Among phenotype pairs from the genotype-driven cross-phenotype associations, we evaluated penetrance in correlation analysis using a clinical database. We focused on the application of PheWAS in order to make it robust and to aid the derivation of biological meaning post-PheWAS. This comprehensive analysis of PheWAS results based on a health check-up database will provide researchers and clinicians with a panoramic overview of the networks among multiple phenotypes and genetic variants, laying groundwork for the practical application of precision medicine.

## Introduction

From the healthcare perspective, the key concept of precision medicine generally refers to incorporating genetic, lifestyle, environmental, and cultural factors into one’s health status to provide personalized healthcare^1,2^. The phenome-wide association study (PheWAS) is one tool able to fulfill this purpose^3^. PheWAS explores associations among genetic variants and a wide range of traits, including clinical outcomes and lifestyle, and environment^4^.

However, PheWAS, to date has encountered several challenges in practice. First, most PheWASs defined phenotypes using International Classification of Diseases (ICD) terms such as billing codes or phecodes (a type of ICD code grouping). These billing codes can bring an underlying bias into healthcare practices^5,6^. Second, most genetic association studies have been done in limited, non-Asian populations^6^. A PheWAS performed on a homogeneous population from a singular nation can be more powerful as the pools of cases and controls are divided across the same populations. Though recent studies have involved Asian populations, such as a PheWAS study in the Japanese population^7^ and construction of an Asian reference genome dataset^8^, only a few studies have been conducted in Asian populations, and no PheWAS has compared the ethnical differences. Third, in general, the final reports of a PheWAS are mainly comprised of data-driven analysis and its results, including a multitude of phenotypes and statistical numbers; as a consequence, expanded application of the results through post-PheWAS secondary analysis is essential. While PheWAS incorporates a variety of phenotypes and the associations are provided in a collectively integrated manner that provides good perspective on the holistic view of a system, it is difficult to understand the meaning for particular diseases or phenotypes.

**Figure 1:**
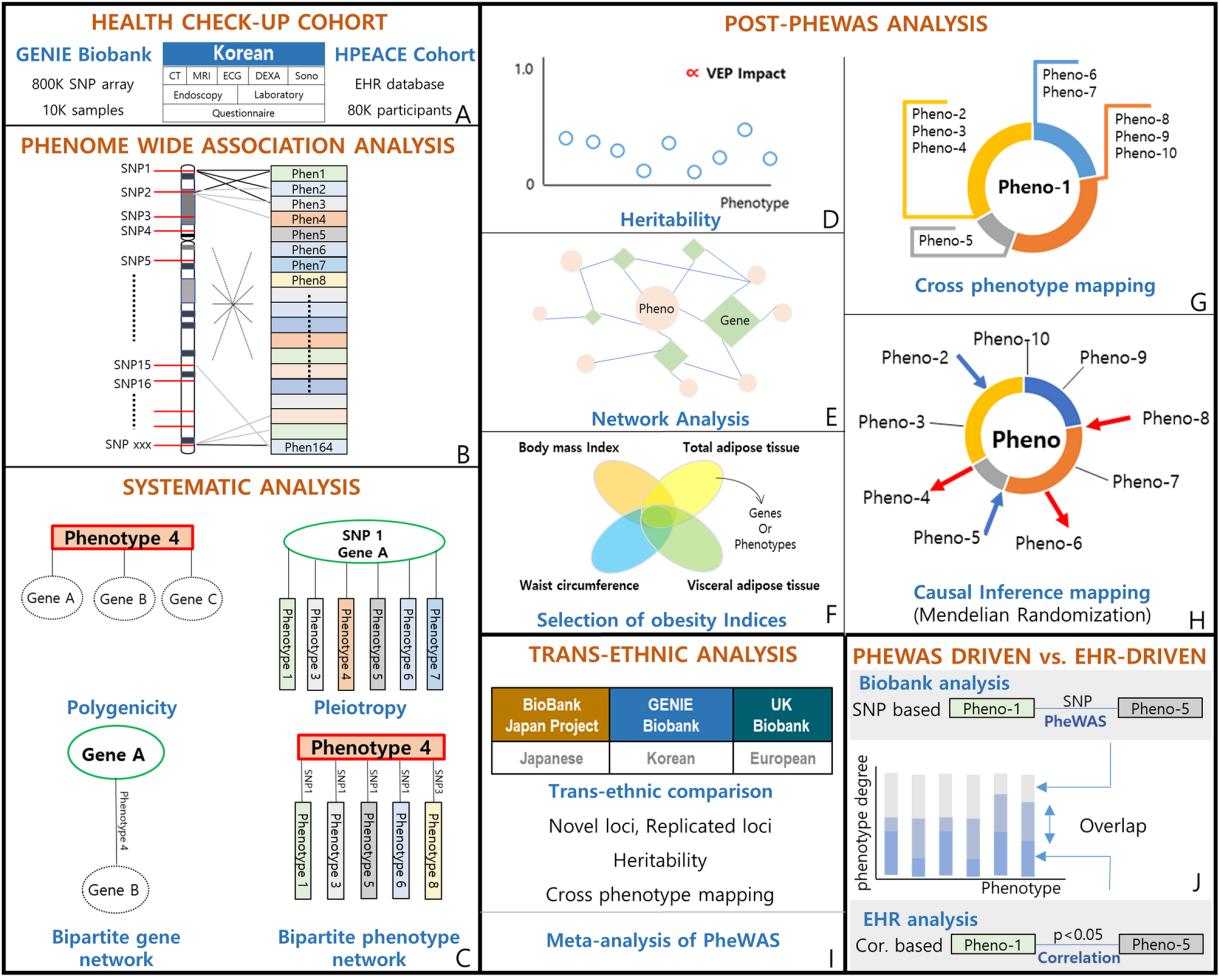
Overview of the study design. (**A**) We utilized a health check-up cohort on comprehensive health check-up. Sub-cohorts of this cohort are the Gene-EnvironmeNtal IntEraction and phenotype (GENIE) cohort, which includes biobank data, and the Health and Prevention EnhAnCEment (H-PEACE) cohort, which includes an EHR database of the health check-up results. (**B**) Phenome wide association study (PheWAS) was performed for 136 phenotypes adjusting for age, sex, and PC1-PC3. (**C**) We leveraged cross-phenotype associations to perform systematic analysis of the PheWAS results, which were polygenicity, pleiotropy, a bipartite gene network, and a bipartite phenotype network. The details are described in Fig. 4. (**D**) To ensure robustness of the PheWAS results, we further dissected the results to suggest applicable interpretations, the heritability for each phenotype; Correlation between phenotype heritability and the effect of the loci on genes and protein sequences associated with phenotypes. (**E**) Using cross-phenotype association information, we constructed phenotype-phenotype and phenotype-genotype networks. (**F**) We visualized the comparison of obesity indices (body mass index, waist circumference, visceral adipose tissue, and total adipose tissue amount). (**G**) We constructed cross-phenotype mappings, which have a core phenotype (Pheno-1 in the figure) and branches of connected phenotypes that share loci. These were partitioned by color according to the biological system involved. (**H**) We estimated causal inferences in the phenotype pairs from cross-phenotype associations using Mendelian randomization and constructed a causal inference map. (**I**) We performed trans-ethnic and trans-nationality analysis among Korean, European, and Japanese populations. (**J**) We compared phenotype-phenotype pairs generated from SNP-based cross phenotype-association in the Biobank analysis with those generated from correlation analysis in the EHR-based H-PEACE cohort. We evaluated the overlap or exclusiveness of pairs for each phenotype by phenotype degree.

In this study, we addressed these challenges by performing a PheWAS in a Korean population based on the deep phenotyping of a health check-up database. This comprehensive health check-up database merged with a biobank and specific to a Korean population is an unprecedented and unique database, making our PheWAS different from those previously. We compared our PheWAS results with results from the UK Biobank (UKBB)^9^ and Biobank Japan project (BBJ)^7^. We also leveraged cross-phenotype associations to perform systematic analyses of the PheWAS results. To ensure the robustness of the results, we further dissected them to expand its application and lay groundwork to derive the biological meaning post-PheWAS (Fig. 1).

The results of this work will provide researchers and clinicians with a panoramic overview of the connections among phenotypes based on genetic associations and allow them to understand healthcare in the perspective of precision medicine.

## Subjects and methods

### Gene-environment of interaction and phenotype (GENIE) cohort

In this study, we used data from the Gene-Environment of Interaction and phenotype (GENIE) cohort, and the Health and Prevention Enhancement (H-PEACE) cohort, at the Seoul National University Hospital Healthcare System Gangnam Center, where comprehensive health check-ups and screening are done in Korean populations. The details of the cohort have been described previously^10^, and is shown in Supplementary Appendix. Definitions of the phenotypes are shown in Table S1.

### Ethics statement

The Institutional Review Board (IRB) of the Seoul National University Hospital approved the biorepository with informed consent (IRB number 1103-127357, Seoul National University Hospital Healthcare System Gangnam Center human Biorepository project). We retrospectively collected the clinical and genetic data, for which the IRB approved this study protocol (IRB number 1706-055-858, Genome-phenome wide association study (PheWAS) using health check up clinical information—genetic database) and waived additional informed consent. All the methods were performed in accordance with relevant guidelines and regulations.

### Genotype data quality control and imputation

At the time of this study, a total of 10,349 individuals had been genotyped using the Affymetrix Axiom KORV 1.0–96 Array (Thermo Fisher Scientific, Santa Clara, CA, USA) by DNA Link, Inc. See the Supplementary Appendix, Table S2, and Figs. S1, S2, S3 for a detailed description of the quality control and imputation process.

### Phenotype data

From the comprehensive health check-up database, we manually collated 65 phenotypes as categorical case/control outcomes and 71 phenotypes as continuous numeric outcomes. Tests corroborative of the 136 phenotypes were abdominal/coronary CT scan, brain MRI/MRA, abdominal ultrasonography, esophagogastroduodenoscopy, fundoscopy, tonometry, electrocardiography, bone mineral densitometry (dual-energy x-ray absorptiometry, DEXA), blood/urine test, spinal X-ray, body composition analyzer (InBodyⓡ), and questionnaire interview (participant reported phenotypic data). The phenotypes were systematized into 13 biological categories according to the body system involved: anthropometric measure (AM), cerebro-cardio-vascular (CV), digestive system (DS), endocrine and metabolism (EM), hematologic system (HS), lifestyle (LS), mental and emotional (ME), minerals (MN), musculoskeletal (MC), ophthalmic system (OS), pulmonary system (PS), renal system (RS), and tumor marker (TM). Detailed information on the phenotypes, such as their definitions, categories, associated data formats, and associated tests, are provided as a glossary in Table S1. An overview of the phenotypes is given in Table 1.

**Table 1:** Overview of the studied phenotypes.

Category	Phenotype	Significant loci count (*p* < 1 × 10^–4^)	Significant loci count (*p* < 4.916 × 10^–10^)	Significant gene count (*p* < 1 × 10^–4^)	Heritability (h^2^)
AM	Anthropometric measure				
AM	Height	2415	5	257	0.3221
AM	Weight	995	0	132	0.2292
AM	Body mass index	886	0	142	0.2375
AM	Skeletal muscle mass	1479	0	192	0.2769
AM	Body fat mass	1135	0	144	0.1995
AM	Body fat percent	1324	0	149	0.2142
AM	Waist circumference	841	0	129	0.1781
AM	Total adipose tissue area	1251	0	128	0.1505
AM	Visceral adipose tissue area	981	0	131	0.1082
CV	Cerebro-cardio-vascular				
CV	Heart rate	1318	40	130	0.1681
CV	Axis on EKC	959	0	142	0.1496
CV	EKG: Sinus bradycardia	862	40	99	0.1062
CV	EKG: Right bundle branch block	786	0	141	0
CV	EKG: 1st degree atrioventricular block	894	0	156	0.0119
CV	EKG: Myocardial infarction	853	0	160	0.0915
CV	EKG: Myocardial ischemia	1459	0	276	0.2081
CV	Coronary CT: Coronary calcium score	2688	19	629	0.1278
CV	Coronary CT: Coronary vascular plaque	1241	0	114	0
CV	Coronary CT: Coronary vascular stenosis	654	0	102	0
CV	Coronary CT: Aortic dilatation	619	0	127	0.1247
CV	Brain unidentified bright object (UBO)	519	0	92	0.1272
CV	Brain small vessel disease	789	0	117	0.0202
CV	Brain vascular atherosclerosis	521	0	105	0.1204
CV	Brain vascular stenosis	901	0	182	0.1987
CV	Brain aneurysm	720	0	111	0.147
CV	Brain atrophy	1246	0	166	0.2294
CV	Diagnosed of hypertension	1039	0	138	0.1024
DS	Digestive system				
DS	Gall bladder adenomyomatosis	817	0	140	0.0733
DS	Pancreas IPMN	873	0	164	0.0875
DS	Liver hemangioma	714	4	121	0.0003
DS	Gall bladder cholecystitis	836	1	156	0.0232
DS	Gall bladder stone	765	0	135	0.0276
DS	Gall bladder polyp	904	1	122	0.1163
DS	Fatty liver	849	144	111	0.1332
DS	Atrophic gastritis	610	0	103	0.015
DS	Intestinal metaplasia of stomach	1074	0	151	0.1527
DS	Duodenal ulcer	833	54	106	0
DS	Gastric ulcer	1000	0	200	0.0315
DS	Gastroesophageal reflux disease	565	0	101	0.0143
DS	Serum total protein	945	52	203	0.1993
DS	Serum albumin	1310	21	231	0.2325
DS	Serum total bilirubin	2570	1151	137	0.274
DS	Alkaline phosphatase	2631	299	203	0.1203
DS	Glutamic oxaloacetic transaminase	2209	8	462	0.0334
DS	Glutamic pyruvic transaminase	1266	6	255	0.0609
DS	Gamma-Glutamyl Transferase	2716	78	512	0.0818
DS	Gastric cancer	982	0	207	0.1719
DS	Hepatitis B virus surface antigen	3762	324	252	0.1679
DS	Hepatitis C virus antibody	1119	0	231	0.0809
EM	Endocrine and metabolism				
EM	Fasting blood glucose level	1842	0	212	0.1116
EM	Uric acid	3977	1261	284	0.2186
EM	Triglycerides	2676	333	258	0.1385
EM	HDL cholesterol	2036	442	171	0.2471
EM	Hemoglobin A1c	1861	0	245	0.1084
EM	Free T4	1652	2	194	0.1547
EM	Thyroid-Stimulating Hormone	15,064	741	2549	0.1016
EM	Total cholesterol	1061	17	151	0.0678
EM	LDL cholesterol	1279	63	142	0.0367
EM	Metabolic syndrome	811	2	132	0.1583
EM	Thyroid cancer	836	0	225	0.0023
EM	Breast cancer	952	0	210	0.0522
EM	Diagnosed of diabetes	1507	0	187	0.0824
EM	Diagnosed of dyslipidemia	1103	2	150	0.1251
HS	Hematologic system				
HS	White blood cell count	1629	143	153	0.1454
HS	Platelet count	3040	185	278	0.2375
HS	Neutrophil percent among WBC	2080	250	200	0.1423
HS	Lymphocyte percent among WBC	1978	247	190	0.1524
HS	Monocyte percent among WBC	1948	18	194	0.2067
HS	Eosinophils percent among WBC	3109	11	343	0.2822
HS	Basophils percent among WBC	4043	293	373	0.2941
HS	Red blood cell count	1997	209	181	0.2582
HS	Hemoglobin	1707	12	199	0.1854
HS	Mean corpuscular volume	3270	250	251	0.2444
HS	Mean corpuscular hemoglobin	3077	134	358	0.2204
HS	Mean corpuscular hemoglobin concentration	4982	1266	979	0.1389
HS	Plateletcrit	2747	68	299	0.2023
HS	Mean Platelet Volume	3843	188	591	0.1353
HS	Prothrombin time	4515	227	846	0.061
HS	Activated Partial Thromboplastin Time	2092	691	181	0.1725
HS	Hematocrit	962	0	170	0.1544
HS	Red blood cell distribution width	2489	146	244	0.1575
LS	Life style				
LS	Smoking history	939	0	99	0.062
LS	Alcohol consumption	2158	612	156	0.0908
LS	Exercise amount	1657	1	357	0
LS	Education level	558	0	134	0.0264
LS	Marital status	0	0	0	0.0048
LS	Coffee consumption	680	17	109	0.0317
LS	Nocturia per night	652	0	98	0.0339
ME	Mental and emotion				
ME	Sleep onset latency	503	0	112	0.0931
ME	Wake Time After Sleep Onset	869	0	121	0.066
ME	Depressed mood	1043	0	163	0.1041
ME	Appetite change increase	808	0	114	0
ME	Diminished cognitive functioning	918	0	152	0.0769
ME	Worthlessness or guilty feeling	1033	0	104	0.0399
ME	Suicidal ideation	1334	0	285	0.2812
ME	Loss of interest or pleasure	993	0	160	0.169
ME	Fatigue	888	0	158	0.046
ME	Psychomotor retardation	869	0	149	0.0698
ME	Psychomotor agitation	1093	0	188	0.0663
ME	Depression score	839	0	141	0.0614
MN	Minerals				
MN	Calcium level	3792	597	763	0.2503
MN	Phosphorus level	1461	1	177	0.106
MN	Sodium level	3992	776	821	0.1384
MN	Potassium level	632	0	94	0
MN	Chloride level	763	3	213	0.0967
MN	CO2 level	759	0	116	0.0499
MN	Vitamin D3	1271	8	117	0.0743
MC	Musculoskeletal				
MC	Bone density by DEXA	799	0	88	0.2982
MC	Spondylosis	419	0	73	0
MC	Spondylolisthesis	939	0	147	0.4245
MC	Compression fracture	1189	0	229	0.4589
MC	Intervertebral disc space narrowing	529	0	97	0.0603
OS	Ophthalmic system				
OS	Cataract	865	0	98	0.0214
OS	Drusen	842	0	124	0
OS	Macular change	881	0	137	0.0347
OS	Optic disc cupping	755	0	133	0.0856
OS	Optic nerve fiber loss	886	0	144	0.1659
OS	Intraocular pressure, right	1468	30	263	0.156
OS	Intraocular pressure, Left	1451	4	175	0.1074
PS	Pulmonary system				
PS	Forced vital capacity (L)	1519	0	188	0.2408
PS	Forced vital capacity (%)	1524	0	192	0.2426
PS	First second of forced expiration (L)	1474	0	182	0.2895
PS	First second of forced expiration (%)	2066	0	168	0.2876
PS	FEV1/FVC	1611	86	228	0.2055
PS	Pulmonary function test category	563	0	95	0.081
RS	Renal system				
RS	Blood Urea Nitrogen	2551	123	450	0.1825
RS	Renal stone	824	0	143	0.1145
RS	Creatinine	2059	29	399	0.2535
RS	Estimated glomerular filtration rate	1353	34	207	0.2791
RS	Urine pH	3432	651	772	0.1166
RS	Urine albumin	1388	0	212	0.1625
TM	Tumor marker				
TM	Cancer Antigen 125	7923	145	1508	0
TM	Carbohydrate antigen 19–9	27,140	936	4624	0.0312
TM	Alpha Fetoprotein	4244	119	654	0.1803
TM	Carcinoembryonic antigen	1835	202	375	0.0356
TM	Prostate-Specific Antigen	12,999	279	2559	0.1082

### Statistical and computational analyses

#### Phenome-wide association study

We used PLATO^11^ to run logistic regression analysis on 65 categorical outcomes and linear regression analysis on 71 continuous outcomes, incorporating 6,860,342 genetic variants in an additive model. We included age, sex, and the first three principal components to adjust for any potential confounding bias due to these variables. To identify significant results, we implemented multiple test correction through LD-aware Bonferroni correction. The conventional Bonferroni test assumes that the association tests for all SNPs are independent and thus divides the alpha by the total number of tests. For our study, instead of correcting *p*-values with the total number of SNPs, we use LD pruning to identify independent SNPs^12^. The threshold we used for association between SNPs was r^2^ = 0.3, which is provided by Sobota et al. for the East Asian population^13^. We established genome-wide significance at *P* < 4.92 × 10^–10^.

Further exploratory analyses were performed using the associated 260,923 loci with a less stringent *P* < 1 × 10^–4^. Though we used the LD pruning method for Bonferroni correction, the *p*-value was still stringent. Thus, in addition to analyzing associations with a stringent *p*-value cutoff, this exploratory threshold allowed us to further expand the boundaries of research by involving a much wider PheWAS landscape^12^.

To perform systematic analysis of the PheWAS results, we leveraged cross-phenotype associations, in which one locus is associated with multiple phenotypes^14^. Such associations include polygenic inheritance, where a phenotype is influenced by more than one gene^15^ (Fig. S4A); and pleiotropy, where a locus or a gene affects more than one phenotype^16^ (Fig. S4B). To further explore and understand polygenicity and pleiotropy, we constructed two networks: a bipartite phenotype network, connecting phenotypes that shared at least one locus^14^ (Fig. S4C) and a bipartite gene network, connecting genes that shared at least one phenotype^14^ (Fig. S4D). In these connections or networks, the degree property indicates the number of direct connections between one core component and other components. For each core gene/phenotype, the number of genes associated or connected with it is defined as its gene degree, and the number of phenotypes associated or connected is its phenotype degree.

We used the cross-phenotype association information to construct a phenotype-phenotype network and a phenotype-genotype network in order to find hidden relationships among phenotypes or genotypes and also to identify hub genes or hub phenotypes. The Gephi software (https://gephi.org/) was used to visualize the network^17^.

#### Gene annotations

We mapped the variants to genes using Ensembl Variant Effect Predictor (VEP)^18^ annotations (RefSeq). By default, VEP annotates variants in 5000 bp upstream and downstream. So, the variants in 5000 bp regions were mapped to the nearest genes.

#### Functional annotations (*p* value < 1 × 10^–4^)

We mapped genetic associations using VEP^18^ in order to annotate the functional relevance of significant loci. Using the VEP annotation, we classified the biological consequences of loci in coding regions (stop-gained variant, slice acceptor variant, splice donor variant, and missense variant) and in non-coding regions. We also annotated UKBB and BBJ variants with VEP to conduct trans-ethnic and trans-national comparisons as described in a later section.

#### Estimated heritability

To determine the contributions of genetic variants to the risk of certain phenotypes, we estimated the heritability of each phenotype. We estimated heritability using LD Score regression with LDSC (version 1.0.1)^19^ on summary statistics from the PheWAS for all phenotypes. For this analysis, we used the East Asian LD Scores from 1000 Genomes as reference LD Score, which served as the independent variable in the LD Score regression (ref-ld-chr) and regression weights (w-ld-chr). General instructions and the East Asian LD Scores from 1000 Genomes are provided here: https://github.com/bulik/ldsc.

#### Comparison in different populations

To compare results across diverse populations, we performed a trans-ethnic comparison utilizing PheWAS results from a European population and a trans-national comparison utilizing results from a Japanese population. For European population, data from the UK Biobank (UKBB)^9^ was used; for the Japanese population, data from the Biobank Japan Project (BBJ)^7^ was used. We downloaded the summary statistics and estimated heritability results of the phenotypes of these results from the following URLs: http://www.nealelab.is/uk-biobank/ and http://jenger.riken.jp/en/result. We tabulated lists of the phenotypes in the UKBB and BBJ and searched for those that were most similar to phenotypes in our database. The manually curated overlapping phenotypes among GENIE, UKBB, and BBJ are given in Table S3.

#### Mendelian randomization

To better understand the causal inferences in cross phenotype mapping, we performed Mendelian randomization (MR) analysis on the phenotype pairs connected in the bipartite phenotype network. To avoid potential bias due to sample overlap between exposure and outcome, we split our dataset into two equal sets by random assignment of samples. PheWAS was conducted on each dataset separately to generate the summary statistics that were used as input to MR. Additionally, significant SNPs (*P* < 1 × 10^–4^) from the initial PheWAS with all samples were used as instrument variables (IV). Furthermore, all IVs that were significant in outcome (*P* < 0.01) were removed, as IVs should not be directly associated with outcome. The SNPs were LD-clumped using very strict cutoff of clump kb = 10,000 and r2 = 0.001. We calculated *p*-values using the inverse-variance weighted (IVW) method from the *MendelianRandomization* package in R^20^. We adjusted for multiple testing using FDR correction. We also performed sensitivity analysis using MR-egger and the median-based method.

#### Meta-analysis of PheWAS

We performed meta-analysis using our PheWAS results and the BBJ results for all phenotypes that were available in both datasets. The BBJ summary statistics came from different studies, requiring harmonization of the files. Phenotype matches between GENIE and BBJ are listed in Table S3. Some of the phenotypes from GENIE matched to multiple phenotypes in BBJ; in such cases, we carried out meta-analysis separately for each BBJ phenotype. The meta-analysis was implemented using METAL^21^. The overall scheme of our study is shown in Fig. 1.

## Results

After QC, the study population of the GENIE cohort included 9742 participants, comprising 5696 males and 4046 females, with average age 50.7 + / − 10.0 years. The characteristics of the study population are given in Table S4.

See the Supplementary Appendix for detailed description of the results.

### Phenome-wide association analysis

From the PheWAS on 136 phenotypes, we found significant associations for 65 phenotypes and 14,101 SNPs (*P* < = 4.92 × 10^–10^). The counts of significant loci and genes associated with each phenotype are given in Table S5 and most significant variants are shown in Tables S6, S7 and Fig. S5. Approximately 1% of variants were in coding regions and 98.885% were in non-coding regions (Fig. S6, Tables S8, S9).

**Figure 2:**
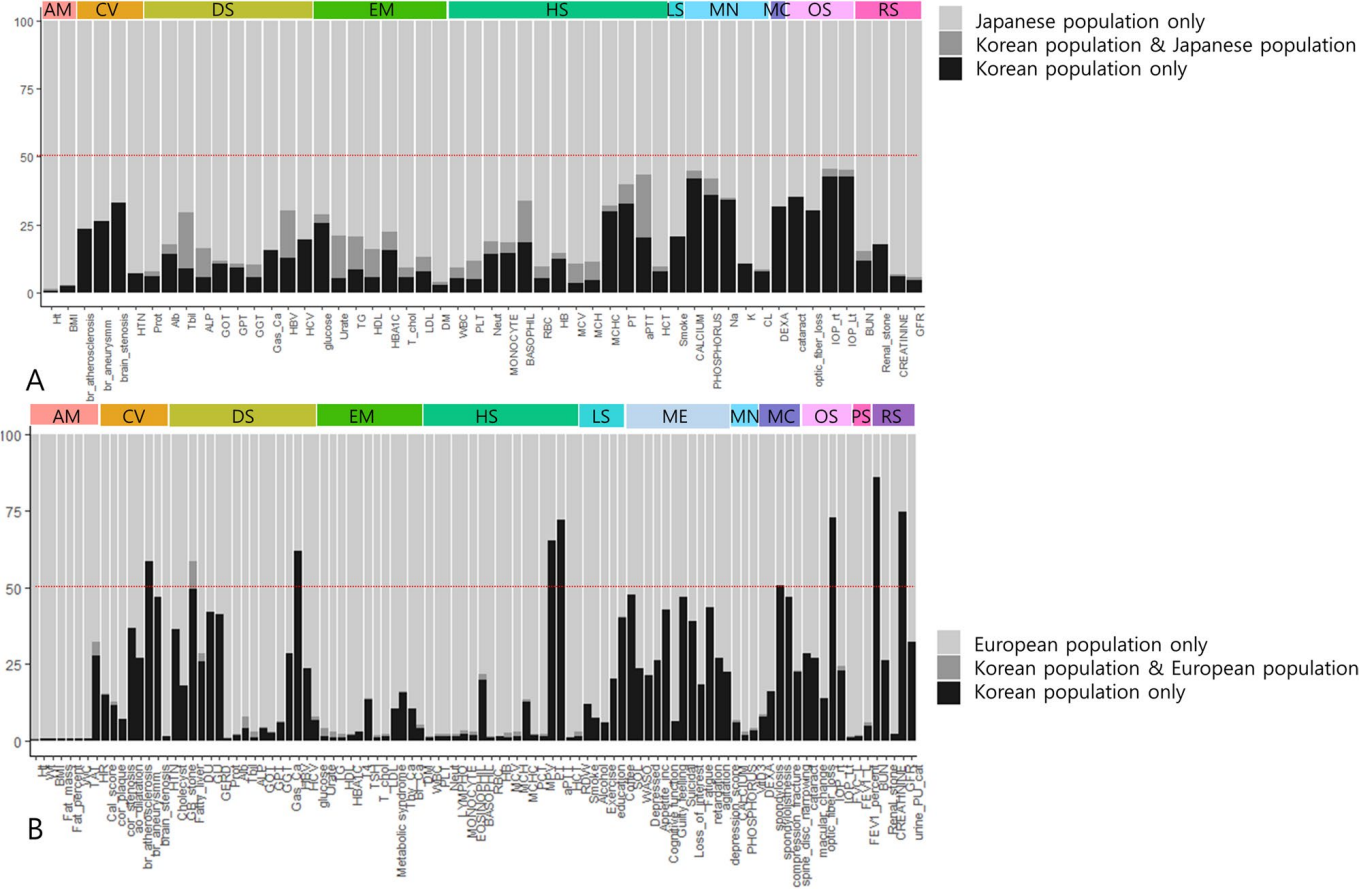
Trans-ethnic, trans-nationality comparison of PheWAS. We compared PheWAS results among Korean, Japanese, and European populations. Phenotypes existing in all datasets were used. We evaluated loci significantly associated only in Koreans (black bar), in both populations (gray bar), and only in the other population (bright gray bar). The colored bar at the top indicates phenotype categories. The Y axis denotes the ratio (%) of loci in each classification, with 100% being the total significant in the compared populations. (**A**) PheWAS result comparison between Korean and Japanese populations. (**B**) PheWAS result comparison between Korean and European populations.

We systematically compared the significant associations of loci and their genes with phenotypes (*P* < 1 × 10^–4^) to results from the BBJ and UKBB to determine if our results were replicated in other populations and also to look for novel findings (Fig. S7, Tables S10, S11). In the comparison between Korean and UK populations, fewer overlapping loci were identified, with the highest overlap ratio being 9.15% in fatty liver disease; 42 phenotypes did not have any overlap (Fig. 2, Fig. S8).

Population comparisons were further investigated for body mass index (BMI) in particular. 34 genes were unique in our populations relative to both Japanese and European populations (Table S12, Fig. S9). Of those unique genes, 23 have previously reported associations with obesity or body weight; the corresponding literature review and references are given in Table S13. The other 11 genes have not been previously reported as associated with obesity in humans, and could be candidate novel genes for BMI or obesity. The details of the genes are described in the Supplementary Appendix.

### Systematic analysis of the PheWAS results

To perform a systematic analysis of the PheWAS results, we leveraged cross-phenotype associations, where one locus is significantly associated with multiple phenotypes. For this analysis, significant loci were filtered by a less-stringent threshold, *P* < 1 × 10^–4^. The schematic structure for this analysis is shown in Fig. S4. Possible polygenicity (Fig. S4A, Table S14); possible pleiotropy (Fig. S4B, Table S15); bipartite phenotype network (Fig. S4C, Table S16); and a bipartite gene network (Fig. S4D) were drawn from PheWAS results.

The bipartite phenotype network comprised 23,580 loci (2902 genes) with 135 phenotypes. There were 1926 distinct pairs of phenotypes. We calculated the degree properties of core phenotypes in this network (Table S17), where core phenotypes were those nodes connected to several phenotypes by shared variants (Fig. S4C). Notably, phenotypes in the tumor markers category had relatively high degree of phenotype connection. Meanwhile, the highest possible polygenicity was observed for mean corpuscular hemoglobin concentration (MCHC), with 782 genes.

The bipartite gene network comprised 14,907 genes, which were connected through sharing associations with the same phenotypes. Table S18 give the gene degree and phenotype degree values for this network. The three genes with the highest phenotype degrees were; CUB and Sushi Multiple Domains 1 Protein (*CSMD1*)*,* RNA-binding Fox-1 Homolog 1 (*RBFOX1*), and Protein Tyrosine Phosphatase Receptor Type D (*PTPRD*); this could be due to possible pleiotropy.

We compared the bipartite phenotype networks of the GENIE (Korea), BBJ (Japanese), and UKBB (European) cohorts. Fig. S10 visualizes the phenotype-phenotype pairs observed in each population; 288 pairs were simultaneously observed in all three populations (Table S19). Notably, these included the pairing of red blood cell count (RBC) and brain vascular atherosclerosis. There are reports of RBC having relation to coronary artery disease^22^ and stroke mortality^23^, but not directly to brain vascular atherosclerosis .

### Secondary analysis of the PheWAS results

#### Heritability analysis

Heritability was calculated for each of the 136 phenotypes by regression of LD scores (Table S20). The top heritability values were obtained for compression fracture, spondylolisthesis, and height. In terms of biological categories and body systems, the highest heritability value was obtained for the musculoskeletal system (Table S21).

The Ensembl variant effect predictor (VEP) provides information regarding the effect of loci on genes and protein sequences (https://useast.ensembl.org/Help/Glossary?id=535). We divided the significant loci (1 × 10^–4^) into two groups according to their annotated impacts, namely “modifier low” vs. “moderate, high”, and evaluated the correlation between impact group and heritability in each phenotype. A significant correlation was observed (*P* = 0.001, correlation (r) = 0.281, 95% CI = 0.117–0.429).

We further compared the heritability in our population with that in the Japanese and European populations (Table S20). Comparisons to each of the Japanese and UK populations are shown in Fig. S11, while the three-way comparison among Korean, Japanese, and UK populations is shown in Fig. S12. The prothrombin time (PT) and activated partial thromboplastin time (aPTT), which are biomarkers of coagulation function, showed similar trends in the Korean and Japanese populations, but manifested relatively high heritability in Koreans relative to the UK population.

#### Network analysis

Using cross-phenotype association information, we constructed phenotype-phenotype and phenotype-genotype networks.

**Figure 3:**
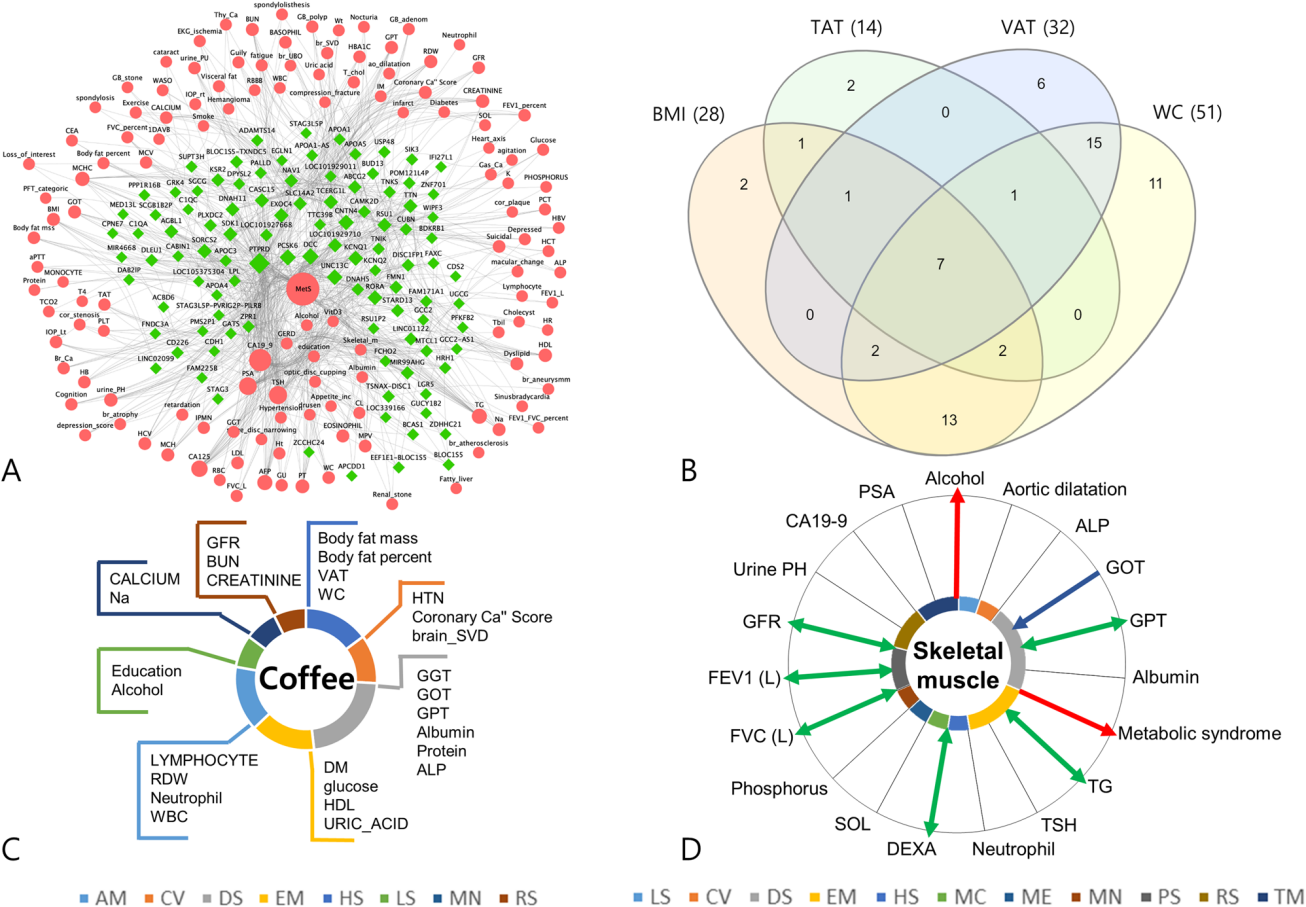
Post-PheWAS analysis. (**A**) *Network analysis* A network representation of gene-phenotype associations related to metabolic syndrome was constructed from 102 genes associated with metabolic syndrome and 128 phenotypes sharing those genes. Each edge is a phenotype-gene association, with genes for significant loci (*P* < 10^–4^) being annotated by VEP. Node size is proportional to degree, which is the number of connections. Pink nodes correspond to phenotypes and green nodes to genes. (**B**) *Relationships among obesity indices* We visualized the comparison among the obesity indices such as body mass index (BMI), waist circumference (WC), visceral adipose tissue (VAT) and total adipose tissue (TAT) amount by drawing a the venn-diagram for cross phenotype association of phenotypes or genes. (**C**) *Cross-phenotype mapping* Cross-phenotype mappings were generated based on the bipartite phenotype network, in turn constructed from the connections among phenotypes sharing at least one locus. Coffee consumption, which is one of the lifestyle phenotypes, had 31 phenotype degrees in the bipartite phenotype network. (**D**) *Causal inference mapping* We estimated causal inferences in phenotype pairs based on cross-phenotype associations using Mendelian randomization (MR), and constructed a causal inference map. The direction of the arrow is the causality result from MR (Blue arrows, skeletal muscle mass as outcome; Red arrows, skeletal muscle mass as exposure; Green arrows, bidirectional). Pairs observed in the bipartite phenotype network but insignificant in MR have straight black lines without arrows.

First, a network representation of gene-phenotype associations related to metabolic syndrome was constructed (Fig. 3A). 132 genes associated with metabolic syndrome and 128 phenotypes sharing 102 genes with metabolic syndrome were used to construct the network. In the metabolic syndrome sub-network, five genes had high degrees of connection and could be considered hub genes: PTPRD, DCC Netrin 1 Receptor (DCC), Proprotein Convertase Subtilisin/kexin Type 6 (PCSK6), Unc-13 Homolog C (UNC13C), and Contactin 4 (CNTN4). The phenotypes in this network comprised: of cardiovascular diseases, of metabolic diseases, used as markers for obesity, and other various disease. The phenotypes in this network comprised: of cardiovascular diseases, of metabolic diseases, used as markers for obesity, and other various disease. The phenotype nodes included triglyceride (TG), HDL cholesterol (HDL), hypertension, diabetes, and waist circumference (WC). These results give a genetic rationale for the definition of metabolic syndrome in the PheWAS perspective.

We also constructed a phenotype-phenotype network using 1,926 phenotype pairs based on shared loci (*P* < 1 × 10^–4^). Fig. S13 shows the phenotype-phenotype network for the whole dataset, and an interactive visualization tool of the phenotype-phenotype network is available (https://hdpm.biomedinfolab.com/ddn/genie/).

#### Relationships among obesity indices

Obesity is a disease entity, which the interest in and research into, has been growing^24,25^. However, definitions of pathological obesity make inconsistent use of variable traits such as body mass index (BMI), waist circumference (WC), total adipose tissue area (TAT), and visceral adipose tissue area (VAT). The defining parameter for obesity also varies between researchers and with respect to the target disease. We visualized^26^ the overlap or exclusiveness among BMI, WC, TAT, and VAT based on the bipartite phenotype network and pleiotropy potential of genes. As shown in Fig. 3B, connections were observed as quadrant intersections among BMI, WC, TAT, and VAT for seven phenotypes: CA19-9, GOT, GPT, body fat mass, body fat percent, weight, and metabolic syndrome. There were 15 phenotypes connected exclusively with VAT and WC, of which, most were crucial intermediate phenotypes that link obesity with diseases. Accordingly, it can be postulated that when defining obesity, VAT or WC would better represent the characteristics of pathogenic obesity. The two genes that are exclusively overlapped between VAT and WC (Fig. S14) could be candidate genes for explaining the pathogenic role of obesity (Table S22).

#### Cross-phenotype mapping

Cross-phenotype mappings were generated based on the bipartite phenotype network, in which the connected phenotypes shared at least one locus.

First, we constructed a cross-phenotype mapping focused on tumor markers. Table S23 shows the respective connected phenotypes we obtained for tumor markers. Fig. S15 shows the cross-phenotype mapping for CEA, which could be considered during oncological practice in order to take into consideration all the possible effects of phenotypes other than colorectal cancer progression itself.

Second, we constructed a cross-phenotype mapping focused on lifestyle factors. In this study, we visualized the cross-phenotype mapping for the coffee consumption. Coffee consumption had 27 phenotypes connected through sharing of significant loci (Fig. 3C). The results of these cross-phenotype mappings could provide the genetic background to explain interactions between environmental factors and disease, and might further provide basic knowledge necessary to conduct gene-environment interaction analysis.

#### Mendelian randomization analysis

We estimated the causal inferences in phenotype pairs based on cross-phenotype associations using Mendelian randomization (MR) (Table S24). As shown in Fig. 3D, we drew a causal inference mapping centered on skeletal muscle mass. The Mendelian randomization analysis yielded nine significant phenotypes, of which one was causal for skeletal muscle mass, two phenotypes were outcomes from skeletal muscle mass, and six had bidirectional relationships with skeletal muscle mass. This analysis revealed that skeletal muscle mass was a significant causal factor for metabolic syndrome and alcohol consumption.

We also performed Mendelian randomization with a focus on lifestyle factors that were causal exposures in cross-phenotype associations, such as alcohol consumption, coffee consumption, exercise amount, and smoking history (Table S25). Alcohol consumption was a significant causal exposure for ten phenotypes, coffee consumption for three phenotypes, exercise amount for six phenotypes, and smoking history for two phenotypes. Coffee consumption was also a significant causal exposure for three anthropometric measurements: body fat mass, visceral adipose tissue area, and waist circumference.

#### Comparison of the phenotype-phenotype pairs between PheWAS-driven versus EHR-driven

**Figure 4:**
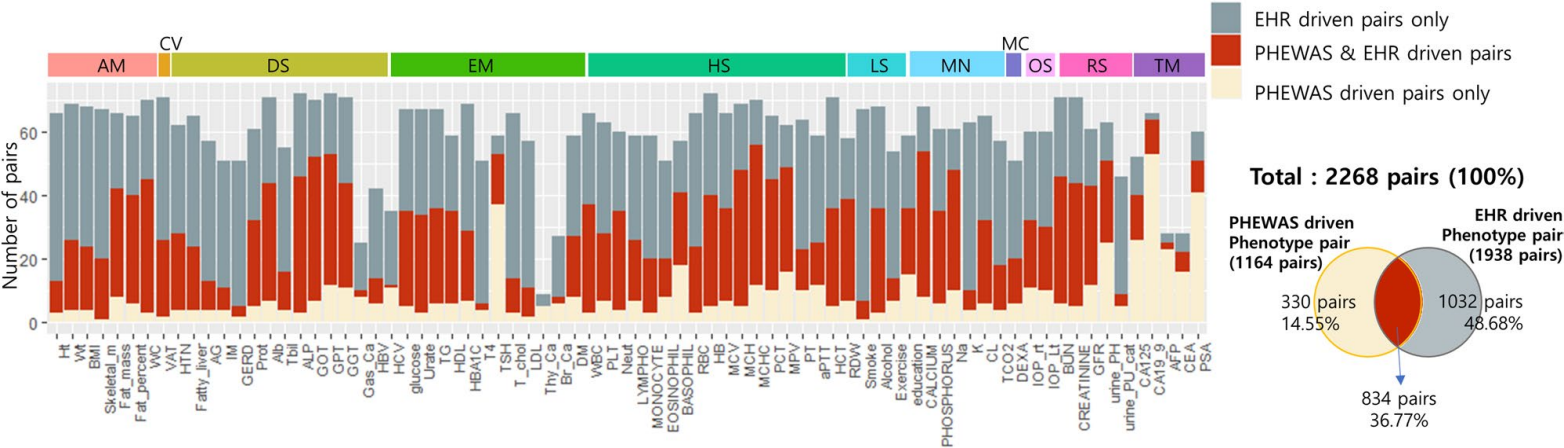
Comparison of phenotype-phenotype pairs between PheWAS driven and EHR-driven analysis. There were 76 phenotypes also recorded in the EHR-driven cohort (H-PEACE cohort). PheWAS-driven pairs (1164) were based on shared SNPs with association *P* < 1 × 10^–4^, and EHR-driven pairs (1938) on correlation analysis with multi-test corrected *P* < 0.05 (Table S26). Skeletal muscle mass (95%) and alkaline phosphatase (93.48%) had high ratios of overlap, while thyroid cancer (0%) and alpha fetoprotein (8%) had low ratios. In terms of biological categories, the average replication % was highest for anthropometric measurement (86.43%) Of the 1164 pairs from the PheWAS-driven approach, 834 (71.65%) also manifested significance in the EHR-driven analysis.

“Penetrance” in genetics is the proportion of those individuals carrying a certain genetic variant who also exhibit the associated phenotype, while “expressivity” measures the proportion of individuals that are carriers of a certain variant and show the associated phenotype to a certain extent^27^. As an indirect method to investigate the penetrance or expressivity of the significant loci identified in our study, we repeated bipartite phenotype network construction using an electronic health records (EHR)-driven method in H-PEACE cohort. Among the phenotypes used in PheWAS analysis, 76 phenotypes were also recorded for this cohort. PheWAS-driven pairs (1164 pairs) were selected based on shared SNPs with association *P* < 1 × 10^–4^, and EHR-driven pairs (1938 pairs) were selected based on correlation analysis with multi-test corrected *P* < 0.05. We compared these phenotype-phenotype pairs (Table S26) and evaluated the overlap or exclusiveness of the pairs for each phenotype. Of the 1164 pairs identified in the PheWAS-driven approach, 834 (71.65%) also manifested significance in the EHR-driven analysis. As shown in Fig. 4 and Table S27, high ratios of overlap were identified for skeletal muscle mass (95%) and alkaline phosphatase (93.48%), and low ratios for thyroid cancer (0%) and alpha fetoprotein (8%). When viewed in terms of biological category, the highest average % replication was obtained for anthropometric measurement (86.43%).

#### Meta-analysis of PheWAS from Korean and Japanese populations

We performed a PheWAS meta-analysis by incorporating our data with the BBJ data (Japanese population). The results are given in Table S28, Figs. S16 and S17. All 51 phenotypes used in the meta-analysis had an increased number of significant variants in the Korean population, while 37 phenotypes had variants uniquely significant in the meta-analysis. Furthermore, height, diabetes and body mass index had more than 100 variants that were uniquely identified as significant in the meta-analysis.

## Discussion

With the advancements in healthcare research that are being driven by big data, increasing efforts are being made to carry out data-wide association studies. PheWAS is one of the tools in that paradigm. However, previous studies faced major challenges in terms of deep phenotyping due to generally using ICD codes, which have limited clarity in their definitions; making the results robust by expanding its application; and the characteristics of population genetics, being highly affected by race and ethnicity. Here, we carried out PheWAS in a Korean population using comprehensive health check-up data linked with genotype data, and furthermore aimed to derive the biological meaning by performing secondary analysis of the PheWAS results. We also compared the results of PheWAS studies conducted in different populations to evaluate trans-ethnic differences. Finally, our bipartite phenotype network analysis of phenotypes using shared genetic association revealed hidden patterns between phenotypes.

The deep phenotypes we used in our studies were corroborated during comprehensive health check-up by various confirmatory methods such as laboratory tests, endoscopy, CT scans, MRI, interview questionnaires, and so on. For each participant, all tests were done in the same institute and on the same day. This process of generating deep phenotypes makes for data quality that is well controlled and consistent when compared to results from phenotypes based on ICD codes, which can be discrepant with actual clinical diagnoses due to biases in billing pattern^28^. As we were able to use the raw data produced by the test, our analysis included a lot of endophenotypes. Endophenotype (intermediate phenotype) is a quantitative biological trait^29^ that is reported to reliably reflect the function of the categorical biological system^29,30^ and has reasonable heritability^31^. As such, an endophenotype could be more closely related to the genetic basis and cause of a clinical trait than would be a broad clinical phenotype such as an ICD code^32^.

We compared our PheWAS results with studies done in European (UK Biobank) and Japanese (Biobank Project Japan) populations and found several novel loci, replicated loci, replicated phenotype-phenotype pairs. We furthermore compared estimated heritability among the populations. Significant variants in the Korean population were partly replicated in both European and Japanese populations, though the replication rate was higher in the Japanese population. We also identified SNP-phenotype associations that were unique to the Korean population when compared to not only the European but also the Japanese population. Noticeably, in the comparison of significant variants associated with body mass index (BMI), the Korean population had novel unique variants (Fig. S8) associated with *TERF2IP, ATRNL1*, and *BANF1*. The results from these trans-ethnic and trans-nationality comparisons seemingly emphasize the importance of considering genetic differences among ethnicities, and also race. Koreans are generally included in the East Asian population; however, study of the human Y-chromosome^33^ suggests that compared to other populations from Asia, the Korean population has characteristics of a distinct, mostly endogamous ethnic group, and living in a confined peninsula area has preserved these monogenic nationality traits. In a study comparing genetic structure and divergence among Han Chinese, Japanese, and Korean populations those three East Asian populations were shown to have distinct genetic make-up and could be distinguished based on their genetic characteristics^34^. In the meta-analysis of our population and the Japanese population, 72.5% of phenotypes had variants that were uniquely significant in the meta-analysis. Our study shows that the common and exclusive genetic associations of phenotypes should be taken into consideration when performing a population-based clinical study. Furthermore, meta-analysis of PheWAS studies in populations of the same ethnicity but different nationalities can discover uniquely significant variants.

In the comparison of the estimated heritability among different populations, the heritability in the Korean population of biomarkers for coagulation function, such as PT and aPTT, showed similar trends with that of the Japanese population, but manifested relatively high heritability when compared to the UK population. This indicates that the contribution of genetic variants to variation in coagulation traits is affected by ethnical differences. Evaluating heritability difference by ethnicity will be important supportive information in the development of drugs as an aspect of precision medicine.

We also leveraged the cross-phenotype association results to provide a panoramic overview of the network connections among multiple phenotypes and genetic variants. Specifically, we generated a phenotype-genotype network focused on metabolic syndrome (Fig. 3A). Metabolic syndrome is a cluster of metabolic abnormalities that are known to be associated with visceral adipose obesity^35^. A large number of epidemiological studies have been conducted on metabolic syndrome because it is a crucial target for healthcare, imposing an increased risk of developing conditions such as cardiovascular disease^35^, malignant disease^36^, depression^37^, and metabolic disease^35^. Early diagnosis is important to prevent the negative consequences of metabolic and this may be done by modifying the lifestyle and risk factors. The network we constructed provided a rationale for defining metabolic syndrome by phenotypes of TG, HDL, hypertension, diabetes, and WC, and for using the characteristics of metabolic syndrome to collectively integrate heterogeneous and complex disease status. The network included phenotypes of cardiovascular disease (coronary calcium score, cardiac ischemia, brain atherosclerosis, malignant disease (thyroid cancer, gastric cancer), and depression and metabolic disease (fatty liver, uric acid), which are known to be complications of metabolic syndrome. Other phenotypes in the network related to obesity, specifically visceral obesity indicator and visceral fat amount; obesity is a well-known cause of metabolic syndrome^35^. Furthermore, lifestyle factor phenotypes such as alcohol consumption, smoking habit, and exercise amount were also part of the network. These suggest modifiable targets for preventing the complications of metabolic syndrome. Finally, the network suggested hub genes associated with metabolic syndrome. Similar network analysis of PheWAS results might provide genotype-based evidence of connections among phenotypes or variants, which to date have been assumed from epidemiological research, and can also provide novel insights into connections that have not been previously reported or recognized.

We additionally used the bipartite phenotype network to perform cross-phenotype mapping. Table S23 shows the cross-phenotype mapping constructed for tumor markers. Tumor markers are highly used in clinical practice for tasks such as oncological screening and monitoring recurrence after treatment. The marker carcinoembryonic antigen (CEA) is recommended by the National Comprehensive Cancer Network (NCCN) guidelines for colon cancer and American Society of Clinical Oncology (ASCO) to test a diagnosis of colon cancer as a baseline for monitoring and then to regularly monitor for recurrence or metastasis of the colon cancer^38,39^. Testing for the marker PSA is recommend by the American Cancer Society (ACS) for men aged > 50 years, after an informed decision-making process^40^. Regular testing for another marker, serum alpha-fetoprotein (AFP), is recommended by the NCCN guideline in the follow-up of hepatocellular carcinoma^41^.

However, while testing for tumor markers is essential in the surveillance of malignant disease, their usage faces problems in the form of low sensitivity and specificity and the potential that they could be affected by factors other than the cancer itself. Thus, providing a cross-phenotype mapping for tumor markers could support an oncologist in interpreting the results of each tumor marker test. For instance, hemoglobin was included in our CEA cross-phenotype mapping. Thus, if a colorectal cancer patient has severe anemia, we should be cautious about interpreting a change in CEA; the anemia could attenuate or exaggerate its reflection of the patient’s cancer status^42^. There are several reports that have used not only one tumor marker but a combination of tumor markers to monitor malignancies^43–45^. In Table S23, each tumor marker has pairs with multiple other tumor markers, which provide supporting evidence for combining tumor markers as a means to improve their utility in malignancy surveillance.

We also built cross-phenotype mappings for environmental factors. Figure 3C shows the cross-phenotype mapping for coffee consumption in particular. Similar visualization of the correlations between environmental factors and other phenotypes could provide insight into which disease should be considered for the investigation of the benefits or hazards of given environmental factors, and what also connections could provide a candidate model for gene x environment interactions.

In our study, we applied Mendelian randomization analysis to cross-phenotype networks in order to generate corresponding causal inference networks. To the best of our knowledge, this is the first approach to utilize MR in network-based analysis. MR enables the estimation of causal inference by evaluating the relationship between genetic susceptibility to the causal factor and the outcome in question^22^. As shown in Fig. 3D, we specifically drew a causal inference map for skeletal muscle mass. We visualized this map because skeletal muscle mass is regarded as an endocrine and paracrine organ, and is also suggested as a marker in diseases such as metabolic syndrome, diabetes, and more^46^. The analysis revealed skeletal muscle mass as having significant causal inference for metabolic syndrome. In the network, Skeletal muscle mass had six bidirectional associations. Bidirectional association means the “A” phenotype could cause “B”, and at the same time “B” phenotype could cause “A”, whether is in forward or reverse way^47,48^. Skeletal muscle mass had a bidirectional association with pulmonary function (FEV1, FVC). There are several epidemiological studies for this association^49–51^. In one of the studies, individuals with reduced skeletal muscle mass amount have caused a decrease in FVC and FEV1, because of weakened ability to inflate and deflate their lungs^49^. In another study, patients with chronic obstructive pulmonary disease (COPD) are a risk factor for skeletal muscle atrophy by complex combination of various pathophysiological alteration leading to suboptimal muscle work^50^. Though the effect of sarcopenia on pulmonary function is mainly emphasized in clinical practice, muscle recovery measures in poor pulmonary function patients should also be well understood. By the information provided by the bidirectional association network, it will raise alerts for researchers to focus on the reversed direction of causality, which is not well reported, by referring to our results. Thus, by performing MR, we can suggest which phenotype could be causal or an outcome in relation with a trait and also begin to elucidate the mechanism or pathophysiology for a disease of interest.

There are a couple of limitations for the Mendelian randomization (MR) analysis results. First, since there was high dimensional degree of significant association pairs (1767 significant pairs by threshold FDR < 0.05), we were not able to provide externally replicated analysis results. For increased confidence, all the significant results in the networks warrants further examination and replication in an external cohort. However, it was difficult to find a cohort with various deep phenotypes, especially those uniquely measured in comprehensive health check-up. For instance, in Fig. 3D, the core phenotype of the network is skeletal muscle amount. Though there are genetic studies regarding sarcopenia52, we couldn’t find any cohort that simultaneously had skeletal muscle mass summary statistics as well as pulmonary function or bone mineral density test summary statistics results. But, as our study utilized a comprehensive health check-up cohort, we were able to evaluate the association between various phenotypes from apparently unrelated body systems. Second, there were several MR associations, difficult to be explained by currently reported epidemiological studies. We were able to provide epidemiologic evidence for some of the associations in Fig. 3D, such as skeletal muscle amount with metabolic syndrome46; pulmonary function49-51; liver function53; and bone mineral density54. But there were several MR associations, which are not biologically explainable. For instance, causal associations between PSA and gastric cancer; right and left intraocular pressure; exercise and hepatitis C virus carrier; skeletal muscle amount and alcohol consumption. There should be several reasons for these findings. First, in the analysis, end-phenotypes, endo-phenotypes and environmental phenotypes are altogether incorporated in the MR analysis. Gene by environmental interaction was not considered in the analysis. Second, our cohort is from the health check-up cohort, which could contain samples with positive morbidity relatively few. Third, we did not have an external validation population but analyzed in one sample population. This could have led to spurious correlations between phenotypes that are unrelated to genetics. These limitations should be further analyzed by performing gene by environment interaction; future analysis in a larger set of study population; and in an external validation study population.

Though the MR analysis results are not validated in other cohorts and several significant associations were not biologically reasonable, it could raise a necessity to validate certain associations by other researchers to focus on certain pairs of phenotypes and organize a cohort for that purpose. In our study, we provide an openly accessible web-based phenotype-phenotype network for the whole dataset (Fig. S13), which is an interactive visualization tool of the phenotype-phenotype network (https://hdpm.biomedinfolab.com/ddn/genie). This tool will allow other researchers to easily access our results and pick up where the unreported associations or limitations are and build up future research.

Our study has several advantages. First, to the best of our knowledge, this is the first PheWAS study performed in the Korean population. As described above, several loci in this population differ from the Japanese population. We were also able to carry out trans-nationality analysis for the PheWAS. Second, we defined phenotypes directly using results from health check-ups and questionnaire responses from personal participants. This makes the resolution, clarity, and reliability of this study’s results better than those of a PheWAS based on ICD codes or personal self-reports. These billing codes or personal memory can bring an underlying bias into the data registry. So, the phenotypes are objectively and precisely defined by these check-ups. Third, since all tests were performed in the same institute, under the same conditions, and by using the same machines, protocols, and chemicals, the produced data is consistent and its quality is highly controlled. Fourth, we performed secondary analysis of the PheWAS results in ways to derive the biological meaning, so that the results could be highly applicable and utilized more practically. We constructed a phenotype-phenotype network using all the phenotypes in our study (Fig. S13). Similarly constructing a phenotype-phenotype network based on comprehensive, deep phenotypes could provide clinicians and researchers with a detailed landscape of the interconnections between phenotypes and enable better understanding of their underpinnings. Furthermore, the phenotype-phenotype network not only includes disease status but also contains information on genes, environment, and lifestyle. Precision medicine pursues prevention and treatment strategies that take individual variability^1^, such as in genes, environment, and lifestyle, into account^2^. Accordingly, the networks generated by PheWAS would provide fundamental information for realizing precision medicine. Fifth, we provide summary statistics, which are significant. This will help other researchers to explore the phenotypes for making headway in further study.

Our study has several limitations. First, we did not have a set of Korean replication population because it was not possible to find such datasets with the variety of deep phenotypes incorporated in our study. However, we instead introduced the UKBB and BBJ as replication sets, and consequently identified multiple replicated loci. We also replicated the phenotype-phenotype pairs using a larger EHR-driven database of Korean samples to investigate whether the genetic connection was reflected at the actual phenotype level. Second, the study population was collected from those who had regular health check-ups, and therefore samples with positive morbidity were relatively few. Accordingly, the significance of the loci was low for some phenotypes. We tried to overcome this lack of statistical power by performing a meta-analysis with the UKBB and BBJ summary statistics, in which we were able to pick up additional significant loci. In a future study, we will incorporate diverse disease cohorts from the Korean population to increase the study power. Third, phenotype-phenotype networks were constructed from a single sample, because it was not possible to find an external set of population. This might have led to spurious correlations between phenotypes that are unrelated to genetics. Further, for most of our analysis we used suggestive *p*-value cutoff of 10^–4^, and there were 260,922 variants that passed the threshold across the phenotypes. However, if FDR < 0.05 cutoff is considered, the number is lower with 114,677 passing the threshold (Table S5), which could have led to inclusion of more false positive associations. Forth, in network analysis, it was based on a permissive p-value threshold, which can be associated with false-positive associations.

In conclusion, our study highlights the capacity for understanding the biological insights post-PheWAS by comprehensively exploiting the results. With the information generated by PheWAS, we attempted to provide a landscape that integrated an individual’s genetic, lifestyle, and environmental factors along with health status. We provided several samples of actionable applications such as constructing a gene-phenotype association network related to metabolic syndrome; constructing cross-phenotype mappings; and visualizing causal inference mappings. Through analysis in the context of differences in ethnicity and nationality, our study shows that some phenotypes are common or exclusive in their genetic associations, and this should be taken into consideration when performing a population-based clinical study. The paradigm of PheWAS suggested in our study will eventually be the cornerstone for applying the core concepts of precision medicine to research and healthcare practice.

## Supplementary Information

Supplementary Information 1.

Supplementary Information 2.

Supplementary Information 3.

Supplementary Information 4.

Supplementary Information 5.

## Data Availability

Complete summary statistics of the GENIE cohort are not publicly available due to restrictions (institutional policy to protect the privacy of research participants), but are available from the corresponding author on reasonable request. However, all other data are contained in the article and its supplementary information or are available upon reasonable request. The summary statistics from UK biobank and Biobank Japan are available at from the following URLs: http://www.nealelab.is/uk-biobank/ and http://jenger.riken.jp/en/result. The codes used in analysis for this paper is available at https://github.com/dokyoonkimlab.

## References

[1] Collins FS, Varmus H (2015). A new initiative on precision medicine. N. Engl. J. Med..

[2] Gonzalez-Hernandez G, Sarker A, O'Connor K, Greene C, Liu H (2018). Advances in text mining and visualization for precision medicine. Pac. Symp. Biocomput..

[3] Denny JC, Bastarache L, Roden DM (2016). Phenome-wide association studies as a tool to advance precision medicine. Ann. Rev. Genomics Hum. Genet..

[4] Denny JC (2013). Systematic comparison of phenome-wide association study of electronic medical record data and genome-wide association study data. Nat. Biotechnol..

[5] Roden DM (2017). Phenome-wide association studies: a new method for functional genomics in humans. J. Physiol..

[6] Hebbring SJ (2014). The challenges, advantages and future of phenome-wide association studies. Immunology.

[7] Kanai M (2018). Genetic analysis of quantitative traits in the Japanese population links cell types to complex human diseases. Nat. Genet..

[8] GenomeAsia KC (2019). The GenomeAsia 100K Project enables genetic discoveries across Asia. Nature.

[9] Bycroft C (2018). The UK Biobank resource with deep phenotyping and genomic data. Nature.

[10] Lee C (2018). Health and prevention enhancement (H-PEACE): a retrospective, population-based cohort study conducted at the Seoul National University Hospital Gangnam Center Korea. BMJ Open.

[11] Hall MA (2017). PLATO software provides analytic framework for investigating complexity beyond genome-wide association studies. Nat. Commun..

[12] Verma A (2018). PheWAS and beyond: the landscape of associations with medical diagnoses and clinical measures across 38,662 individuals from geisinger. Am. J. Hum. Genet..

[13] Sobota RS (2015). Addressing population-specific multiple testing burdens in genetic association studies. Ann. Hum. Genet.

[14] Darabos, C., Harmon, S. H. & Moore, J. H. Using the bipartite human phenotype network to reveal pleiotropy and epistasis beyond the gene. *Pac Symp Biocomput*, 188–199 (2014). PMC3900286 24297546

[15] Pazoki R (2018). Methods for polygenic traits. Methods Mol. Biol..

[16] Stearns FW (2010). One hundred years of pleiotropy: a retrospective. Genetics.

[17] Bastian, M., Heymann, S. & Jacomy, M. Gephi: an open source software for exploring and manipulating networks. *International AAAI Conference on Weblogs and Social Media* (2009).

[18] McLaren W (2016). The ensembl variant effect predictor. Genome Biol..

[19] Bulik-Sullivan BK (2015). LD Score regression distinguishes confounding from polygenicity in genome-wide association studies. Nat. Genet..

[20] Yavorska OO, Burgess S (2017). MendelianRandomization: an R package for performing Mendelian randomization analyses using summarized data. Int. J. Epidemiol..

[21] Willer CJ, Li Y, Abecasis GR (2010). METAL: fast and efficient meta-analysis of genomewide association scans. Bioinformatics.

[22] Zheng J (2019). Use of mendelian randomization to examine causal inference in osteoporosis. Front. Endocrinol. (Lausanne).

[23] Hatamian H, Saberi A, Pourghasem M (2014). The relationship between stroke mortality and red blood cell parameters. Iran J. Neurol..

[24] De Lorenzo A (2019). Obesity: a preventable, treatable, but relapsing disease. Nutrition.

[25] Kyle TK, Dhurandhar EJ, Allison DB (2016). Regarding obesity as a disease: evolving policies and their implications. Endocrinol. Metab. Clin. North Am..

[26] Heberle H, Meirelles GV, da Silva FR, Telles GP, Minghim R (2015). InteractiVenn: a web-based tool for the analysis of sets through Venn diagrams. BMC Bioinf..

[27] Taeubner J (2018). Penetrance and Expressivity in Inherited Cancer Predisposing Syndromes. Trends Cancer.

[28] Wei WQ (2017). Evaluating phecodes, clinical classification software, and ICD-9-CM codes for phenome-wide association studies in the electronic health record. PLoS ONE.

[29] Meyer-Lindenberg A, Weinberger DR (2006). Intermediate phenotypes and genetic mechanisms of psychiatric disorders. Nat. Rev. Neurosci..

[30] Flint J, Munafo MR (2007). The endophenotype concept in psychiatric genetics. Psychol. Med..

[31] Cannon TD, Keller MC (2006). Endophenotypes in the genetic analyses of mental disorders. Ann. Rev. Clin. Psychol..

[32] Gottesman II, Gould TD (2003). The endophenotype concept in psychiatry: etymology and strategic intentions. Am. J. Psychiatry.

[33] Kim SH, Han MS, Kim W, Kim W (2010). Y chromosome homogeneity in the Korean population. Int. J. Legal Med..

[34] Wang Y, Lu D, Chung YJ, Xu S (2018). Genetic structure, divergence and admixture of Han Chinese ,Japanese and Korean populations. Hereditas.

[35] Despres JP, Lemieux I (2006). Abdominal obesity and metabolic syndrome. Nature.

[36] Esposito K, Chiodini P, Colao A, Lenzi A, Giugliano D (2012). Metabolic syndrome and risk of cancer: a systematic review and meta-analysis. Diabetes Care.

[37] Pan A (2012). Bidirectional association between depression and metabolic syndrome: a systematic review and meta-analysis of epidemiological studies. Diabetes Care.

[38] Benson AB (2017). Colon Cancer, Version 1.2017, NCCN clinical practice guidelines in oncology. J. Natl. Compr. Cancer Netw..

[39] Locker GY (2006). ASCO 2006 update of recommendations for the use of tumor markers in gastrointestinal cancer. J. Clin. Oncol..

[40] Smith RA (2017). Cancer screening in the United States, 2017: a review of current American Cancer Society guidelines and current issues in cancer screening. CA Cancer J. Clin..

[41] Yu SJ (2016). A concise review of updated guidelines regarding the management of hepatocellular carcinoma around the world: 2010–2016. Clin. Mol. Hepatol..

[42] Kang HY, Choe EK, Park KJ, Lee Y (2017). Factors requiring adjustment in the interpretation of serum carcinoembryonic antigen: a cross-sectional study of 18,131 healthy nonsmokers. Gastroenterol. Res. Pract..

[43] He CZ (2013). Combined use of AFP, CEA, CA125 and CAl9-9 improves the sensitivity for the diagnosis of gastric cancer. BMC Gastroenterol.

[44] Bozkurt M, Yumru AE, Aral I (2013). Evaluation of the importance of the serum levels of CA-125, CA15-3, CA-19-9, carcinoembryonic antigen and alpha fetoprotein for distinguishing benign and malignant adnexal masses and contribution of different test combinations to diagnostic accuracy. Eur. J. Gynaecol. Oncol..

[45] Xu HX (2017). Postoperative serum CEA and CA125 levels are supplementary to perioperative CA19-9 levels in predicting operative outcomes of pancreatic ductal adenocarcinoma. Surgery.

[46] Kim G, Kim JH (2020). Impact of Skeletal Muscle Mass on Metabolic Health. Endocrinol. Metab. (Seoul).

[47] Choi KW (2019). Assessment of bidirectional relationships between physical activity and depression among adults: a 2-sample mendelian randomization study. JAMA Psychiat..

[48] Steinmo S, Hagger-Johnson G, Shahab L (2014). Bidirectional association between mental health and physical activity in older adults: Whitehall II prospective cohort study. Prev. Med..

[49] Park CH, Yi Y, Do JG, Lee YT, Yoon KJ (2018). Relationship between skeletal muscle mass and lung function in Korean adults without clinically apparent lung disease. Med. (Baltim.).

[50] Jaitovich A, Barreiro E (2018). Skeletal muscle dysfunction in chronic obstructive pulmonary disease. What we know and can do for our patients. Am. J. Respir. Crit. Care Med..

[51] Marklund S, Bui KL, Nyberg A (2019). Measuring and monitoring skeletal muscle function in COPD: current perspectives. Int. J. Chron. Obstruct. Pulmon. Dis..

[52] Tan LJ, Liu SL, Lei SF, Papasian CJ, Deng HW (2012). Molecular genetic studies of gene identification for sarcopenia. Hum. Genet..

[53] Altajar S, Baffy G (2020). Skeletal muscle dysfunction in the development and progression of nonalcoholic fatty liver disease. J. Clin. Transl. Hepatol..

[54] Ferrucci L (2014). Interaction between bone and muscle in older persons with mobility limitations. Curr. Pharm. Des..

