# Supplementary material for: Leveraging deep phenotyping from health check-up cohort with 10,000 Korean individuals for phenome-wide association study of 136 traits
Source: Sci Rep. 2022 Feb 4;12:1930. doi: 10.1038/s41598-021-04580-2 (PMC8817039; doi:10.1038/s41598-021-04580-2)
Supplement: Supplementary file 5 — Supplementary Information 5. [file 41598_2021_4580_MOESM5_ESM.pdf]

Leveraging deep phenotyping from health check-up cohort with 10,000 Korean individuals for phenome-wide association study of 136 traits

Supplementary Appendix

▌Subjects and Methods

Gene-Environment of Interaction and phenotype (GENIE) cohort

The Gene-Environment of Interaction and phenotype (GENIE) cohort provides a comprehensive database of biomarkers related to non-communicable diseases, lifestyle, medical history, environmental factors, and individual genetic information. The details of the cohort have been described previously [1]. Briefly, the SNUH Gangnam Center provides comprehensive health check-ups and screening, with nearly 20,000 people visiting this center annually. All participants go through complete questionnaires, physical examinations, laboratory blood and urine tests, abdominal sonography, and gastroscopy. Selectively and on participant request, they also receive advanced tests such as coronary computed tomography (CT), gastroscopy, abdominal CT, and brain magnetic resonance Imaging/magnetic resonance angiography (MRI/MRA). The study population is predominantly Korean. As per consent, we collected blood samples and aliquoted several blood specimens. We also annotated the H-PEACE cohort as an electrical health record (EHR) database of comprehensive health check-ups from the Korean population and the GENIE cohort as a genotype database linked to the EHR database. Further preprocessing was conducted for the information in the EHR database. Free text records and questionnaire answers were manually curated by clinicians based on the definitions shown in Table S1. Logical errors and artifacts in the results were manually probed and corrected.

Genotype data quality control and imputation

At the time of this study, a total of 10,349 individuals had been genotyped using the Affymetrix Axiom KORV 1.0-96 Array (Thermo Fisher Scientific, Santa Clara, CA, USA) by DNA Link, Inc. This array, referred to as the Korean Chip, was designed by the Center for Genome Science, Korea National Institute of Health; optimized for the Korean population; and available through the K-CHIP consortium. A Korean Chip comprises >833,000 markers, including >247,000 rare-frequency or functional variants estimated from the sequencing data of >2,500 Koreans [2].

We performed systematic quality control (QC) on the raw genotype data. SNPs with minor allele frequencies <1%, low marker call rate (<5%), and significant deviation from Hardy-Weinberg equilibrium in controls (HWE < 1e-05) were excluded. Samples with discordant sex info (0.3 < X-chr homozygosity < 0.8, = PROBLEM), low sample call rate (call rate < 0.9, mind 0.1), or extreme heterozygosity (heterozygosity rate > mean +/- 3 SD), along with one individual from any related pairs identified (IBD >= 0.125), were excluded. After quality control was performed, 548,755 SNPs remained. GWAS imputation was carried out using Eagle 2.4.1 (https://data.broadinstitute.org/alkesgroup/Eagle/) and Minimac3 (https://genome.sph.umich.edu/wiki/Minimac3). We used the Northeast Asian Reference Database (NARD) + 1000 Genome Phase 3 database (1KG) re-phased panel as the reference panel and the NARD imputation server (https://nard.macrogen.com) for imputation. NARD [3] includes the whole-genome sequencing data of 1,779 individuals from Korea, Mongolia, Japan, China, and Hong Kong, which are not present in 1KG. We compared the imputation quality of chromosome 22 using different reference panels, such as NARD vs. 1KG vs. NARD + 1KG, and determined that the NARD + 1KG panel had the best accuracy (Table S2). Analysis included only high-quality imputed common SNPs, which were those having minor allele frequency >0.01 and imputation R^2 (Minimac3's r-squared metric) >0.7 (Figure S1). After sample-level QC, genotype-level QC, and imputation, a total of 6,860,342 SNPs from 9,742 individuals were

included in the analysis. LD pruning was done using plink (–show-tags --list-all --tag-r2 0.2 --tag-kb 250) to calculate the degree of loci shared by different phenotypes.

The influence of ethnicity was assessed through analysis of population stratification using principal component analysis (PCA) implemented in EIGENSOFT package v6.1.4. We used the first three principal components (PCs) to adjust for population stratification (Figure S2). The steps by which the raw data was preprocessed are shown in Figure S3.

Supplementary Results

Phenome-wide association analysis for 136 phenotypes

GENIE cohort (Korean population)

From the PheWAS on 136 phenotypes, we found significant associations for 65 phenotypes (50 from continuous variables, 13 from categorical variables) and 14,101 SNPs at $P <= 4.92x10^{-10}$. The counts of significant loci and genes associated with each phenotype are given in Table S5. Among continuous phenotypes, the top five most significant were activated partial thromboplastin time (aPTT), LDL cholesterol, serum total bilirubin, uric acid, and carcinoembryonic antigen (CEA). Among categorical phenotypes, the top five most significant were alcohol consumption, fatty liver, duodenal ulcer, coffee consumption, and Hepatitis B virus surface antigen (Table S6, Figure S5). In the Manhattan plot, aPTT had two top signal loci, in chromosome 5 (rs1801020&CR982412, $P = 2.85x10^{-214}$) and chromosome 9 (rs676996, $P = 8.99x10^{-72}$). Table S7 lists the top five signals from the GWAS results for each phenotype.

We further performed functional annotation for 221,462 unique loci passing the less stringent *p*-value threshold of $1x10^{-4}$ using Ensembl Variant Effect Predictor (VEP) [4]. Approximately 1% of variants were in coding regions and 98.885% were in non-coding regions

(Figure S6), which result is similar to other large-scale PheWAS [5]. In coding regions, this annotation identified 22 stop-gained variants, six splice acceptor variants, ten splice donor variants, and 1103 missense variants (Table S8 and S9).

Among the 22 stop-gained variants, we replicated an association between rs121907892 and uric acid that is a well-reported finding unique to the east Asian population (EAS) [6], including Koreans [7]. We further identified the stop-gained variant rs200340875 to be significantly associated with blood urea nitrogen (BUN; P = 2.75x10^{-07}, beta = -0.373), calcium (P = 7.15x10^{-08}, beta = -0.044), glutamic oxaloacetic transaminase (GOT; P = 9.65x10^{-6}, beta = 1.189), mean corpuscular hemoglobin (MCH; P = 4.93x10^{-7}, beta = 0.21), mean corpuscular hemoglobin concentration (MCHC; P = 8.53x10^{-16}, beta = 0.228), mean platelet volume (MPV; P = 8.06x10^{-7}, beta = -0.088), urine protein (P = 1.16x10^{-10}, beta = -0.135), and sodium (Na; P = 1.92x10^{-15}, beta = -0.371). According to 1000 Genomes, the minor allele of rs200340875 is reported in African populations (AFR) but not in EAS. The locus of this variant is associated with CD109 Molecule (*CD109*), which has been previously reported with diffuse large B-cell lymphoma [8], psoriasis [9], and gallbladder malignancy [10]. Another stop-gained variant identified was rs145035679, which showed a protective effect for CEA (P = 1.07x10^{-8}, beta = -0.166) and increased risk for carbohydrate antigen 19-9 (CA 19-9; P = 5.81x10^{-5}, beta = 2.178); this variant is associated with Fucosyltransferase 6 (*FUT6*). *FUT6* has previously reported associations with pancreatic cancer [11], breast cancer [12], and colorectal cancer [13]. Among splice acceptor variants, rs112911835 was significantly associated with prothrombin time (PT; P = 1.12x10^{-10}, beta = -0.031), while rs112911835 was associated with Long Intergenic Non-Protein Coding RNA 1933 (*LINC01933*), which has no known relationship with disease as of yet. The splice donor variant rs140944893 showed significant association with coronary vessel calcium scoring (P = 4.29x10^{-5}, beta = -127.7), and is associated with Phospholipase D3 (*PLD3*). This gene is reported to be related to Alzheimer's disease in EAS [14] and EUR [15].

Comparison with BBJ (Japanese) and UKBB (European)

We systematically compared the significant associations of loci and their genes with phenotypes ($P < 1 \times 10^{-4}$) to results from the BBJ and UKBB to determine if our results were replicated in other populations and also to look for novel findings. Originally, each population used a different SNP array platform and a variety of different phenotypes. Accordingly, we first filtered the examined loci and phenotypes to determine overlap with our data, then identified replicated and novel genes and loci. The schematic structures of the trans-ethnical and trans-nationality comparisons are shown in Figure S7. We identified 52 phenotypes overlapping the Japanese biobank results (42 phenotypes had replicated loci) and 101 phenotypes overlapping with the UK Biobank results (59 phenotypes had replicated loci). Gene-level and locus-level comparisons are respectively given in Table S10 and Table S11.

In the comparison between Korean and Japanese populations, aPTT and serum total bilirubin had high overlap of significant loci. Among the 4,016 loci significantly associated with aPTT in Koreans or Japanese, 920 loci (22.91%) were significant in both; among the 6,159 loci associated with total bilirubin in those populations, 1,263 (20.51%) likewise overlapped. Notably, loci associated with the ophthalmic system (cataract and optic fiber loss), cerebrovascular system (brain stenosis, aneurysm, and atherosclerosis), smoking habit, hepatitis C virus antibody, renal stone, gastric cancer, and bone mineral density were mutually exclusive between Koreans and Japanese.

In the comparison between Korean and UK populations, fewer overlapping loci were identified, with the highest overlap ratio being 9.15% in fatty liver disease; 42 phenotypes did not have any overlap (Figure 2, Figure S8).

Population comparisons were further investigated for body mass index (BMI) in particular. For this phenotype, 136 loci (0.42% of significant loci) were replicated in the

Japanese population and 105 loci (0.07%) in the European population, respectively. Our population showed 583 exclusive loci (1.82%) when compared to the Japanese population, and 669 exclusive loci (0.45%) when compared to the European population. We then looked more deeply into the BMI genes unique to the Korean population. Relative to the Japanese population, 73 genes were exclusively associated with the Korean population; meanwhile, relative to the European population, 53 genes were exclusively associated with the Korean population. Of these genes, 34 (714 loci) were unique relative to both Japanese and European populations (Table S12, Figure S9). Of those unique genes, 23 have previously reported associations with obesity or body weight; the corresponding literature review and references are given in Table S13. The other 11 genes have not been previously reported as associated with obesity in humans, and could be candidate novel genes for BMI or obesity; these were Vesicle Amine Transport 1-Like (*VAT1L*), Uromodulin-like 1 (*UMODL1*), Telomeric Repeat-Binding Factor 2-Interacting Protein 1 (*TERF2IP*), Proline-rich Transmembrane Protein 3 (*PRRT3*), PRRT3 Antisense RNA 1 (*PRRT3-AS1*), Long Intergenic Non-protein Coding RNA 578 (*LINC00578*), Family with Sequence Similarity 225 Member B (*FAM225B*), Cation Channel Sperm-associated 1 (*CATSPER1*), Barrier To Autointegration Factor 1 (*BANF1*), Attractin-Like Protein 1 (*ATRNL1*), and Adherens Junctions-associated Protein 1 (*AJAP1*). Among those genes, *TERF2IP* is known from a mouse study to have roles in regulating adipose function and excess fat accumulation, and also protecting against obesity [16]. *ATRNL1* has no previous report related to obesity, but Attractin (*ATRN*) has similarity with the mouse mahogany protein, which is involved in controlling obesity [17,18]. *BANF1* has no known direct association for obesity, but it is reported to suppress expression of S100 calcium-binding protein A9 (*S100A9*) [19], which is a candidate marker for obesity in non-type 2 diabetes mellitus [20].

Systematic analysis of the PheWAS results

GENIE cohort (Korean population)

To perform a systematic analysis of the PheWAS results, we leveraged cross-phenotype associations, where one locus is significantly associated with multiple phenotypes. For this analysis, significant loci were filtered by a less-stringent threshold, $P < 1x10^{-4}$ (loci count = 260,922, gene count = 14,907). The schematic structure for this analysis is shown in Figure S4. Briefly, we constructed: “Possible polygenicity”, in which a phenotype is influenced by more than one gene (Figure S4A, Table S14); “Possible pleiotropy”, in which a locus or gene affects multiple phenotypes (Figure S4B, Table S15); a “bipartite phenotype network” based on the connections among phenotypes sharing at least one locus (Figure S4C, Table S16); and a “bipartite gene network” as the connections among genes shared by at least one phenotype (Figure S4D).

Of the 260,922 significant PheWAS loci, the bipartite phenotype network comprised 23,580 loci (2,902 genes) with 135 phenotypes. There were 1,926 distinct pairs of phenotypes. We calculated the degree properties of core phenotypes in this network (Table S17), where core phenotypes were those nodes connected to several phenotypes by shared variants; an example is phenotype 4 in Figure S4C. Notably, phenotypes in the tumor markers category had relatively high degree of phenotype connection. The highest phenotype degree was obtained for a representative tumor marker for pancreas cancer, *CA 19-9*, with 110 phenotypes connected through sharing of significant loci. Meanwhile, the highest possible polygenicity was observed for mean corpuscular hemoglobin concentration (MCHC), with 782 genes.

The bipartite gene network comprised 14,907 genes, which were connected through sharing associations with the same phenotypes. Table S18 give the gene degree and phenotype degree values for this network. The three genes with the highest phenotype degrees were; CUB and Sushi Multiple Domains 1 Protein (*CSMD1*), RNA-binding Fox-1 Homolog 1 (*RBFOX1*), and Protein Tyrosine Phosphatase Receptor Type D (*PTPRD*); this could be due to

possible pleiotropy. The same three genes had the highest gene degree values; gene degree comprises the edges in bipartite gene networks. Notably, *CSMD1* was significantly associated with 58 phenotypes (showing possible pleiotropy) and connected to 12,602 genes through common associations with phenotypes. *CSMD1* has been reported to function as a complement control protein [21]; complement is implicated in many diseases through the mechanisms of inflammation and autoimmunity [22]. In some cancers, it functions as a tumor suppressor gene [23,24].

Bipartite phenotype network comparison with BBJ and UKBB

We compared the bipartite phenotype networks of the GENIE (Korea), BBJ (Japanese), and UKBB (European) cohorts. There were 49 GENIE phenotypes in common among all datasets, which were used to generate the bipartite phenotype network. Figure S10 shows a Venn diagram of the phenotype-phenotype pairs observed in each population; 288 pairs were simultaneously observed in all three populations (Table S19). Notably, these included the pairing of red blood cell count (RBC) and brain vascular atherosclerosis. There are reports of RBC having relation to coronary artery disease [25] and stroke mortality [26], but not directly to brain vascular atherosclerosis.

Secondary analysis of the PheWAS results, Post-PheWAS analysis

Heritability analysis

Heritability was calculated for each of the 136 phenotypes by regression of LD scores (Table S20). The top heritability values were obtained for compression fracture (h2 = 0.459), spondylolisthesis (h2 =0.425), height (h2 = 0.322), and bone mineral density (h2 = 0.298). In terms of biological categories and body systems, the highest heritability values were obtained for the musculoskeletal system (mean h2 = 0.244), the pulmonary system (mean h2 = 0.225),

anthropometric measures (mean h2 = 0.213), and the hematologic system (mean h2 = 0.188) (Table S21). Though spondylolisthesis and spondylosis involve spinal condition, spondylolisthesis (mean h2 = 0.425) had high heritability but spondylosis had very low heritability (mean h2 = 0.000). Spondylolisthesis is defined as displacement of the vertebral body anteriorly, whereas spondylosis involves a defect in the pars interarticularis of the vertebral arch, which is degeneration in spine[27]. It is reported that around 15% of spondylosis patients have progression to spondylolisthesis, which could be postulated that some portion of the spondylosis might have progressed to spondylolisthesis in our study participants[28,29]. Spondylosis is in some part a natural aging process [30]. Since all the participants in our study are adults and the mean age is above 50 years, the prevalence of spondylosis was high (51.3%) compared to spondylolisthesis (3.5%). There are no genetics studies directly comparing the genetic characteristics between spondylosis and spondylolisthesis, because those who take spinal imaging test will be the patient with back pain. In our study, we were able to get the spine images in the mostly asymptomatic population. The difference in heritability might raise the needs to perform research on genetic etiology between these spinal conditions.

The Ensembl variant effect predictor (VEP) provides information regarding the effect of loci on genes and protein sequences, categorized as modifier impact (usually for non-coding variants or variants affecting non-coding genes, where predictions are difficult or there is no evidence of impact), low impact (mostly harmless or unlikely to change protein behavior), moderate impact (non-disruptive variant that might change protein effectiveness), and high impact (high, disruptive impact on the protein) (https://useast.ensembl.org/Help/Glossary?id=535). We divided the significant loci ($1x10^{-4}$) into two groups according to their annotated impacts, namely “modifier low” vs. “moderate, high”, and evaluated the correlation between impact group and heritability in each phenotype. A significant correlation was observed (*P* = 0.001, correlation (r) = 0.281, 95% CI = 0.117-0.429).

We further compared the heritability in our population with that in the Japanese and European populations (Table S20). Of phenotypes that overlapped with ours, BBJ provides heritability for 35 and UKBB for 101. Since the provided heritability values were determined using different loci and methods, we normalized the heritability to make it comparable. Comparisons to each of the Japanese and UK populations are shown in Figure S11, while the three-way comparison among Korean, Japanese, and UK populations is shown in Figure S12 (33 phenotypes overlapped among the three populations). Generally, most phenotypes had similar trends in heritability across populations. Noticeable differences were observed in percent eosinophils among white blood cell counts (BASOPHIL), prothrombin time in the international normalized ratio (PT), and activated partial thromboplastin time (aPTT). BASOPHIL had relatively high heritability in the Korean population relative to both others. Meanwhile, PT and aPTT, which are biomarkers of coagulation function, showed similar trends in the Korean and Japanese populations, but manifested relatively high heritability in Koreans relative to the UK population.

Network analysis

Using cross-phenotype association information, we constructed phenotype-phenotype and phenotype-genotype networks in order to find hidden relationships among phenotypes or genotypes and to discover hub genes or phenotypes.

First, a network representation of gene-phenotype associations related to metabolic syndrome was constructed (Figure 3A). We selected the nodes by filtering for genes associated with metabolic syndrome, which were identified by annotating the significant loci (P < 10-4). Then, we filtered for phenotypes significantly associated with those selected genes. In the process, edges corresponding to loci not annotated by VEP were not included. Ultimately, 132 genes associated with metabolic syndrome and 128 phenotypes sharing 102 genes with metabolic syndrome were used to construct the network (Figure 3A). The nodes were colored

with respect to gene and phenotype, while the edges are associations between phenotypes and genes. In the metabolic syndrome sub-network, five genes had high degrees of connection and could be considered hub genes: *PTPRD*, DCC Netrin 1 Receptor (*DCC*), Proprotein Convertase Subtilisin/kexin Type 6 (*PCSK6*), Unc-13 Homolog C (*UNC13C*), and Contactin 4 (*CNTN4*). The phenotypes in this network comprised: of cardiovascular diseases, of metabolic diseases, used as markers for obesity, and other various disease. The phenotype nodes included triglyceride (TG), HDL cholesterol (HDL), hypertension, diabetes, and waist circumference (WC). These results give a genetic rationale for the definition of metabolic syndrome in the PheWAS perspective.

We also constructed a phenotype-phenotype network using 1,926 phenotype pairs based on shared loci ($P < 1 \times 10^{-4}$). Figure S13 shows the phenotype-phenotype network for the whole dataset, and an interactive visualization tool of the phenotype-phenotype network is available (https://hdpm.biomedinfolab.com/genie/).

Relationships among obesity indices

Ever since the American Medical Association (AMA) declared obesity to be a disease, interest in and research into obesity has been growing [31,32]. However, definitions of pathological obesity make inconsistent use of variable traits such as body mass index (BMI), waist circumference (WC), total adipose tissue area (TAT), and visceral adipose tissue area (VAT). There are reports of an obesity paradox when defining obesity by BMI [33]. The defining parameter for obesity also varies between researchers and with respect to the target disease. To investigate the relationships among these parameters, we constructed Venn diagrams [34] and visualized the overlap or exclusiveness among BMI, WC, TAT, and VAT based on the bipartite phenotype network (phenotype level) and pleiotropy/polygenicity potential of genes (gene level). As shown in Figure 3B, connections were observed as quadrant intersections among BMI, WC, TAT, and VAT for seven phenotypes: CA19-9, GOT, GPT, body fat mass, body fat percent,

weight, and metabolic syndrome. There were 15 phenotypes connected exclusively with VAT and WC, and the intersection between these traits had two exclusive genes associated. Of the 15 phenotypes, most were crucial intermediate phenotypes that link obesity with diseases. Accordingly, it can be postulated that when defining obesity, VAT or WC would better represent the characteristics of pathogenic obesity. The two genes that are exclusively overlapped between VAT and WC (Figure S14) could be candidate genes for explaining the pathogenic role of obesity. The elements in each set are listed in Table S22.

Cross-phenotype mapping

Cross-phenotype mappings were generated based on the bipartite phenotype network, in which the connected phenotypes shared at least one locus.

First, we constructed a cross-phenotype mapping focused on tumor markers. Several tumor markers are used in screening for cancer, monitoring its recurrence, and evaluating its response to interventions. Commonly-used tumor markers include carcinoembryonic antigen (CEA), carbohydrate antigen 19-9 (CA19-9), alpha fetoprotein (AFP), and prostate-specific antigen (PSA); specifically, CEA serves as a marker for colorectal cancer [35], CA19-9 for pancreato-biliary cancer [36], AFP for liver cancer [37], and PSA for prostate cancer [38]. However, the limitation of using the tumor markers is that it can have low sensitivity or specificity [39], such that a test result could be associated with or affected by various non-malignant conditions. For instance, CEA is known to be affected by hemoglobin level [40], and CA19-9 is reported to be elevated in nonmalignant respiratory disease [41]. Table S23 shows the respective connected phenotypes we obtained for tumor markers; among these, CEA is associated with hemoglobin level and CA19-9 with pulmonary function test, which are consistent with previous reports [40,41]. Figure S15 shows the cross-phenotype mapping for CEA, which could be considered during oncological practice in order to take into consideration all the possible effects of phenotypes other than colorectal cancer progression itself.

Second, we constructed a cross-phenotype mapping focused on lifestyle factors. The analyzed phenotypes included lifestyle factors such as coffee consumption and alcohol consumption. Several studies have shown genotype x environment interactions (G x E) in smoking behaviors [42]. In this study, we visualized the cross-phenotype mapping for the coffee consumption as a starting point for G x E study in this phenotype. Coffee consumption had 27 phenotypes connected through sharing of significant loci (Figure 3C). Several reports have documented relationships between coffee consumption and obesity [43], hypertension [44], diabetes [45], renal function [46], and lipid metabolism [47]. The phenotypes connected with coffee consumption in this mapping (Table S16) support the previous reports of clinical association studies. In the mapping for alcohol consumption, 38 phenotypes shared significant loci. Various studies have identified heavy alcohol consumption as a risk factor for renal disease [48] and coronary artery calcification [49]. The results of these and other cross-phenotype mappings could provide the genetic background to explain interactions between environmental factors and disease, and might further provide basic knowledge necessary to conduct G x E analysis.

Mendelian randomization analysis

We estimated the causal inferences in phenotype pairs based on cross-phenotype associations using Mendelian randomization (MR). Table S24 shows the MR results for each pair having false discovery rate (FDR) < 0.05. Of the phenotype pairs, significant in the cross-phenotype association, 1766 retained significant association after the Mendelian randomization analysis. As shown in Figure 3D, we drew a causal inference mapping centered on skeletal muscle mass. The network grid is based on information from the bipartite phenotype network of skeletal muscle mass. We excluded those pairs whose biological categories were anthropometric measurements, which category includes skeletal muscle mass. The Mendelian randomization analysis yielded nine significant phenotypes, of which one was causal for skeletal muscle mass, two phenotypes were outcomes from skeletal muscle mass, and six had

bidirectional relationships with skeletal muscle mass. This analysis revealed that skeletal muscle mass was a significant causal factor for metabolic syndrome and alcohol consumption. Its bidirectional relationships were with bone mineral density, liver function (GPT), pulmonary function (FVC, FEV1), renal function (glomerular filtration rate), and triglyceride.

We also performed Mendelian randomization with a focus on lifestyle factors that were causal exposures in cross-phenotype associations, such as alcohol consumption, coffee consumption, exercise amount, and smoking history. Table S25 shows the significant outcome phenotypes (FDR < 0.05) from this analysis. Alcohol consumption was a significant causal exposure for ten phenotypes, coffee consumption for three phenotypes, exercise amount for six phenotypes, and smoking history for two phenotypes. Coffee consumption was also a significant causal exposure for three anthropometric measurements: body fat mass, visceral adipose tissue area, and waist circumference.

Comparison of the phenotype-phenotype pairs between PheWAS-driven vs. EHR-driven

"Penetrance" in genetics is the proportion of those individuals carrying a certain genetic variant who also exhibit the associated phenotype, while "expressivity" measures the proportion of individuals that are carriers of a certain variant and show the associated phenotype to a certain extent [50]. As an indirect method to investigate the penetrance or expressivity of the significant loci identified in our study, we repeated bipartite phenotype network construction using an electronic health records (EHR)-driven method. This clinical database consisted of 81,086 distinct participants who went through comprehensive health check-ups from 2004 to 2015 in the SNUH Gangnam Center (H-PEACE cohort). The tests and questionnaires included most of the phenotypes used in the PheWAS study; specifically, 76 phenotypes were also recorded for this cohort. PheWAS-driven pairs (1164 pairs) were selected based on shared SNPs with association $P < 1\times10^{-4}$, and EHR-driven pairs (1938 pairs) were selected based on correlation analysis with multi-test corrected $P < 0.05$. We compared these phenotype-

phenotype pairs (Table S26) and evaluated the overlap or exclusiveness of the pairs for each phenotype. Of the 1164 pairs identified in the PheWAS-driven approach, 834 (71.65%) also manifested significance in the EHR-driven analysis. As shown in Figure 5 and Table S27, high ratios of overlap were identified for skeletal muscle mass (95%) and alkaline phosphatase (93.48%), and low ratios for thyroid cancer (0%) and alpha fetoprotein (8%). When viewed in terms of biological category, the highest average % replication was obtained for anthropometric measurement (86.43%).

Meta-analysis of PheWAS from Korean and Japanese populations

We performed a PheWAS meta-analysis by incorporating our data with the BBJ data (Japanese population). The results are given in Table S28, Figure S16 and Figure S17. All 51 phenotypes used in the meta-analysis had an increased number of significant variants in the Korean population, while 37 phenotypes had variants uniquely significant in the meta-analysis. Furthermore, height, diabetes and body mass index had more than 100 variants that were uniquely identified as significant in the meta-analysis.

References

1 Lee, C. *et al.* Health and Prevention Enhancement (H-PEACE): a retrospective, population-based cohort study conducted at the Seoul National University Hospital Gangnam Center, Korea. *BMJ Open* **8**, e019327, doi:10.1136/bmjopen-2017-019327 (2018).

2 Moon, S. *et al.* The Korea Biobank Array: Design and Identification of Coding Variants Associated with Blood Biochemical Traits. *Sci Rep* **9**, 1382, doi:10.1038/s41598-018-37832-9 (2019).

3 Yoo, S. K. *et al.* NARD: whole-genome reference panel of 1779 Northeast Asians improves imputation accuracy of rare and low-frequency variants. *Genome Med* **11**, 64, doi:10.1186/s13073-019-0677-z (2019).

4 McLaren, W. *et al.* The Ensembl Variant Effect Predictor. *Genome Biol* **17**, 122, doi:10.1186/s13059-016-0974-4 (2016).

5 Verma, A. *et al.* PheWAS and Beyond: The Landscape of Associations with Medical Diagnoses and Clinical Measures across 38,662 Individuals from Geisinger. *Am J Hum Genet* **102**, 592-608, doi:10.1016/j.ajhg.2018.02.017 (2018).

6 Hamajima, N. *et al.* Serum uric acid distribution according to SLC22A12 W258X genotype in a cross-sectional study of a general Japanese population. *BMC Med Genet* **12**, 33, doi:10.1186/1471-2350-12-33 (2011).

7 Lee, J. H. *et al.* Prevalence of hypouricaemia and SLC22A12 mutations in healthy Korean subjects. *Nephrology (Carlton)* **13**, 661-666, doi:10.1111/j.1440-1797.2008.01029.x (2008).

8 Yokoyama, M. *et al.* CD109, a negative regulator of TGF-beta signaling, is a putative risk marker in diffuse large B-cell lymphoma. *Int J Hematol* **105**, 614-622, doi:10.1007/s12185-016-2173-1 (2017).

9 Liu, X. X. *et al.* Association of down-regulation of CD109 expression with up-expression of Smad7 in pathogenesis of psoriasis. *J Huazhong Univ Sci Technolog Med Sci* **36**, 132-136, doi:10.1007/s11596-016-1555-1 (2016).

10 Dong, F., Lu, C., Chen, X., Guo, Y. & Liu, J. CD109 is a novel marker for squamous cell/adenosquamous carcinomas of the gallbladder. *Diagn Pathol* **10**, 137, doi:10.1186/s13000-015-0375-0 (2015).

11 Gao, H. F. *et al.* Overexpressed N-fucosylation on the cell surface driven by FUT3, 5, and 6 promotes cell motilities in metastatic pancreatic cancer cell lines. *Biochem Biophys Res Commun* **511**, 482-489, doi:10.1016/j.bbrc.2019.02.092 (2019).

12 Li, N. *et al.* MicroRNA-106b targets FUT6 to promote cell migration, invasion, and proliferation in human breast cancer. *IUBMB Life* **68**, 764-775, doi:10.1002/iub.1541 (2016).

13 Liang, L. *et al.* miR-125a-3p/FUT5-FUT6 axis mediates colorectal cancer cell proliferation, migration, invasion and pathological angiogenesis via PI3K-Akt pathway. *Cell Death Dis* **8**, e2968, doi:10.1038/cddis.2017.352 (2017).

14 Tan, M. S., Zhu, J. X., Cao, X. P., Yu, J. T. & Tan, L. Rare Variants in PLD3 Increase Risk for Alzheimer's Disease in Han Chinese. *J Alzheimers Dis* **64**, 55-59, doi:10.3233/JAD-180205 (2018).

15 Cacace, R. *et al.* Rare Variants in PLD3 Do Not Affect Risk for Early-Onset Alzheimer Disease in a European Consortium Cohort. *Hum Mutat* **36**, 1226-1235, doi:10.1002/humu.22908 (2015).

16 Yeung, F. *et al.* Nontelomeric role for Rap1 in regulating metabolism and protecting against obesity. *Cell Rep* **3**, 1847-1856, doi:10.1016/j.celrep.2013.05.032 (2013).

17 Nagle, D. L. *et al.* The mahogany protein is a receptor involved in suppression of obesity. *Nature* **398**, 148-152, doi:10.1038/18210 (1999).

18 Tang, W. *et al.* Secreted and membrane attractin result from alternative splicing of the human ATRN gene. *Proc Natl Acad Sci U S A* **97**, 6025-6030, doi:10.1073/pnas.110139897 (2000).

19 Wang, S. *et al.* S100A8/A9 in Inflammation. *Front Immunol* **9**, 1298, doi:10.3389/fimmu.2018.01298 (2018).

20 Mortensen, O. H. *et al.* Calprotectin--a novel marker of obesity. *PLoS One* **4**, e7419, doi:10.1371/journal.pone.0007419 (2009).

21 Kraus, D. M. *et al.* CSMD1 is a novel multiple domain complement-regulatory protein highly expressed in the central nervous system and epithelial tissues. *J Immunol* **176**, 4419-4430, doi:10.4049/jimmunol.176.7.4419 (2006).

22 Wong, E. K. S. & Kavanagh, D. Diseases of complement dysregulation-an overview. *Semin Immunopathol* **40**, 49-64, doi:10.1007/s00281-017-0663-8 (2018).

23 Kamal, M. *et al.* Loss of CSMD1 expression disrupts mammary duct formation while enhancing proliferation, migration and invasion. *Oncol Rep* **38**, 283-292, doi:10.3892/or.2017.5656 (2017).

24 Toomes, C. *et al.* The presence of multiple regions of homozygous deletion at the CSMD1 locus in oral squamous cell carcinoma question the role of CSMD1 in head and neck carcinogenesis. *Genes Chromosomes Cancer* **37**, 132-140, doi:10.1002/gcc.10191 (2003).

25 Schaffer, A. *et al.* Impact of red blood cells count on the relationship between high density lipoproteins and the prevalence and extent of coronary artery disease: a single centre study [corrected]. *J Thromb Thrombolysis* **40**, 61-68, doi:10.1007/s11239-015-1174-x (2015).

26 Hatamian, H., Saberi, A. & Pourghasem, M. The relationship between stroke mortality and red blood cell parameters. *Iran J Neurol* **13**, 237-240 (2014).

27 Gagnet, P., Kern, K., Andrews, K., Elgafy, H. & Ebraheim, N. Spondylolysis and spondylolisthesis: A review of the literature. *J Orthop* **15**, 404-407, doi:10.1016/j.jor.2018.03.008 (2018).

28 Hu, S. S., Tribus, C. B., Diab, M. & Ghanayem, A. J. Spondylolisthesis and spondylolysis. *Instr Course Lect* **57**, 431-445 (2008).

29 Wang, C., Tian, F., Zhou, Y., He, W. & Cai, Z. The incidence of cervical spondylosis decreases with aging in the elderly, and increases with aging in the young and adult population: a hospital-based clinical analysis. *Clin Interv Aging* **11**, 47-53, doi:10.2147/CIA.S93118 (2016).

30 Wilmink, J. T. The normal aging spine and degenerative spinal disease. *Neuroradiology* **53 Suppl 1**, S181-183, doi:10.1007/s00234-011-0924-5 (2011).

31 De Lorenzo, A. *et al.* Obesity: A preventable, treatable, but relapsing disease. *Nutrition* **71**, 110615, doi:10.1016/j.nut.2019.110615 (2019).

32 Kyle, T. K., Dhurandhar, E. J. & Allison, D. B. Regarding Obesity as a Disease: Evolving Policies and Their Implications. *Endocrinol Metab Clin North Am* **45**, 511-520, doi:10.1016/j.ecl.2016.04.004 (2016).

33 Barth, R. F., Maximilian Buja, L., Cao, L. & Brodsky, S. V. An Obesity Paradox: Increased Body Mass Index Is Associated with Decreased Aortic Atherosclerosis. *Curr Hypertens Rep* **19**, 55, doi:10.1007/s11906-017-0753-y (2017).

34 Heberle, H., Meirelles, G. V., da Silva, F. R., Telles, G. P. & Minghim, R. InteractiVenn: a web-based tool for the analysis of sets through Venn diagrams. *BMC Bioinformatics* **16**, 169, doi:10.1186/s12859-015-0611-3 (2015).

35 Duffy, M. J. *et al.* Tumor markers in colorectal cancer, gastric cancer and gastrointestinal stromal cancers: European group on tumor markers 2014 guidelines update. *Int J Cancer* **134**, 2513-2522, doi:10.1002/ijc.28384 (2014).

36 Scara, S., Bottoni, P. & Scatena, R. CA 19-9: Biochemical and Clinical Aspects. *Adv Exp Med Biol* **867**, 247-260, doi:10.1007/978-94-017-7215-0_15 (2015).

37 Galle, P. R. *et al.* Biology and significance of alpha-fetoprotein in hepatocellular carcinoma. *Liver Int* **39**, 2214-2229, doi:10.1111/liv.14223 (2019).

38 Pezaro, C., Woo, H. H. & Davis, I. D. Prostate cancer: measuring PSA. *Intern Med J* **44**, 433-440, doi:10.1111/imj.12407 (2014).

39 Sharma, S. Tumor markers in clinical practice: General principles and guidelines. *Indian J Med Paediatr Oncol* **30**, 1-8, doi:10.4103/0971-5851.56328 (2009).

40 Kang, H. Y., Choe, E. K., Park, K. J. & Lee, Y. Factors Requiring Adjustment in the Interpretation of Serum Carcinoembryonic Antigen: A Cross-Sectional Study of 18,131 Healthy Nonsmokers. *Gastroenterol Res Pract* **2017**, 9858931, doi:10.1155/2017/9858931 (2017).

41 Kodama, T., Satoh, H., Ishikawa, H. & Ohtsuka, M. Serum levels of CA19-9 in patients with nonmalignant respiratory diseases. *J Clin Lab Anal* **21**, 103-106, doi:10.1002/jcla.20136 (2007).

42 Do, E. K. & Maes, H. H. Genotype x Environment Interaction in Smoking Behaviors: A Systematic Review. *Nicotine Tob Res* **19**, 387-400, doi:10.1093/ntr/ntw153 (2017).

43 Lee, J., Kim, H. Y. & Kim, J. Coffee Consumption and the Risk of Obesity in Korean Women. *Nutrients* **9**, doi:10.3390/nu9121340 (2017).

44 Rhee, J. J. *et al.* Coffee and caffeine consumption and the risk of hypertension in postmenopausal women. *Am J Clin Nutr* **103**, 210-217, doi:10.3945/ajcn.115.120147 (2016).

45 Santos, R. M. & Lima, D. R. Coffee consumption, obesity and type 2 diabetes: a mini-review. *Eur J Nutr* **55**, 1345-1358, doi:10.1007/s00394-016-1206-0 (2016).
46 Wijarnpreecha, K., Thongprayoon, C., Thamcharoen, N., Panjawatanan, P. & Cheungpasitporn, W. Association of coffee consumption and chronic kidney disease: A meta-analysis. *Int J Clin Pract* **71**, doi:10.1111/ijcp.12919 (2017).
47 Farias-Pereira, R., Park, C. S. & Park, Y. Mechanisms of action of coffee bioactive components on lipid metabolism. *Food Sci Biotechnol* **28**, 1287-1296, doi:10.1007/s10068-019-00662-0 (2019).
48 Cheungpasitporn, W. *et al.* High alcohol consumption and the risk of renal damage: a systematic review and meta-analysis. *QJM* **108**, 539-548, doi:10.1093/qjmed/hcu247 (2015).
49 Yun, K. E. *et al.* Alcohol and coronary artery calcification: an investigation using alcohol flushing as an instrumental variable. *Int J Epidemiol* **46**, 950-962, doi:10.1093/ije/dyw237 (2017).
50 Taeubner, J. *et al.* Penetrance and Expressivity in Inherited Cancer Predisposing Syndromes. *Trends Cancer* **4**, 718-728, doi:10.1016/j.trecan.2018.09.002 (2018).
